# Mucosal multivalent NDV-based vaccine provides cross-reactive immune responses against SARS-CoV-2 variants in animal models

**DOI:** 10.3389/fimmu.2025.1524477

**Published:** 2025-03-17

**Authors:** Irene González-Domínguez, Adam Abdeljawad, Tsoi Ying Lai, Marta Boza, Stephen McCroskery, Nicholas Lemus, Stefan Slamanig, Gagandeep Singh, Prajakta Warang, Temima Yellin, Anass Abbad, Juan Manuel Carreño, Victoria Dolange, Jose Luis Martínez-Guevara, Gagandeep Singh, Marina Barcena-Varela, Lauren A. Chang, Michael Schotsaert, Florian Krammer, Peter Palese, Weina Sun

**Affiliations:** ^1^ Department of Microbiology, Icahn School of Medicine at Mount Sinai, New York, NY, United States; ^2^ Swammerdam Institute for Life Sciences, University of Amsterdam, Amsterdam, Netherlands; ^3^ Global Health Emerging Pathogens Institute, Icahn School of Medicine at Mount Sinai, New York, NY, United States; ^4^ Department of Oncological Sciences, Icahn School of Medicine at Mount Sinai, New York, NY, United States; ^5^ Icahn Genomics Institute, Icahn School of Medicine at Mount Sinai, New York, NY, United States; ^6^ Marc and Jennifer Lipschultz Precision Immunology Institute, Icahn School of Medicine at Mount Sinai, New York, NY, United States; ^7^ Center for Vaccine Research and Pandemic Preparedness (C-VaRPP), Icahn School of Medicine at Mount Sinai, New York, NY, United States; ^8^ Department of Pathology, Molecular and Cell-Based Medicine, Icahn School of Medicine at Mount Sinai, New York, NY, United States; ^9^ Ignaz Semmelweis Institute, Interuniversity Institute for Infection Research, Medical University of Vienna, Vienna, Austria; ^10^ Department of Medicine, Division of Infectious Diseases, Icahn School of Medicine at Mount Sinai, New York, NY, United States

**Keywords:** transmission-proof, sterilizing immunity, viral shedding, COVID-19, Coronavirus, vector vaccine

## Abstract

**Introduction:**

A new generation of mucosal vaccine against the ever-evolving SARS-CoV-2 is of great value to fight COVID-19. In previous studies, our groups developed a viral vector vaccine based on an avirulent Newcastle disease virus (NDV) expressing the prefusion-stabilized spike protein of SARS-CoV-2 (NDV-HXP-S).

**Methods:**

Here we characterized the *in vivo* biodistribution and immunogenicity of a live mucosal NDV-HXP-S vaccine in animal models.

**Results:**

NDV showed restricted replication in mice and hamsters. Despite limited replication, intranasal live NDV-HXP-S provided protection against SARS-CoV-2 challenge and direct-contact transmission in hamsters. Importantly, a trivalent live NDV-HXP-S vaccine (Wuhan, Beta, Delta) induced more cross-reactive antibody responses against the phylogenetically distant Omicron variant than the ancestral vaccine. Furthermore, intranasal trivalent live NDV-HXP-S boosted systemic and mucosal immunity in mice pre-immunized with mRNA vaccine.

**Discussion:**

Overall, a mucosal multivalent live NDV-HXP-S vaccine shows great promise as a safe, next-generation vaccine conferring broad mucosal and systemic immunity against future SARS-CoV-2 variants.

## Introduction

Severe acute respiratory syndrome coronavirus 2 (SARS-CoV-2) is the causative agent of COVID-19 (Coronavirus Disease 2019). Since the beginning of the pandemic, the development of an effective vaccine has been a major effort. A successful vaccine must be safe and protective, but also cost-effective and accessible on a global scale. Furthermore, with the constant emergence of new SARS-CoV-2 variants of interest (VOIs), the vaccine needs either to be easily updated or to be able to confer broad protection. Although mRNA vaccines have been widely used in the US and the European Union [Our World in Data, as of September 6^th^ 2023 (1)], a different scenario has occurred worldwide where cheaper vaccines dominated the market during the first vaccination campaigns (as of December 14^th^ 2021) (2).

In previous work, our groups have developed a highly immunogenic vaccine based on Newcastle disease virus (NDV) as a viral vector expressing the prefusion-stabilized spike protein from SARS-CoV-2 (3). NDV-HXP-S (hexa proline-spike) can be produced at large-scale and at low-cost through the utilization of existing manufacturing facilities for influenza virus vaccines (4). NDV-HXP-S has proven to work as an intramuscular systemic vaccine and as a mucosal vaccine when administered intranasally (3). The safety and immunogenicity of the ancestral NDV-HXP-S vaccine has been tested in mice, hamsters, pigs and rats (3, 5, 6), and NDV-HXP-S has been used in clinical trials as a mucosal vaccine in the US (Mount Sinai, NCT05181709) or as a systemic vaccine (delivered intramuscularly) in Thailand (NCT04764422, HXP-GPOVac) (7), Vietnam (NCT04830800, COVIVAC) (8), Brazil (NCT04993209) and Mexico (NCT04871737, Patria) (9, 10). Although the safety profile of live NDV has been well studied as a human oncolytic vector and as a veterinary vaccine (11–13), less is known about the viral shedding of NDV after vaccination. To improve the breadth of protection, our previous study demonstrated that a multivalent formulation based on the inactivated NDV-HXP-S variant vaccines (Wuhan, Beta, Delta) given intramuscularly can confer a better protective immune response than the ancestral vaccine alone against a future Omicron variant (14).

In this work, we aim to study the tissue distribution, immunogenicity and *in vivo* protection against a homologous and heterologous SARS-CoV-2 variants of a live mucosal NDV-HXP-S vaccine in preclinical animal models. We used the ancestral NDV-HXP-S vaccine to study its biodistribution and its ability to induce mucosal immunity and to reduce viral transmission. Next, we characterized the multivalent formulation of the live NDV-HXP-S variant vaccine given via the mucosal route in terms of its breadth of protection against the mismatched Omicron BA.1 SARS-CoV-2 strain as an important addition to our previous study on the inactivated vaccine (15). We tested the immunity of this proof-of-principle mucosal multivalent vaccine in both naïve and pre-immunized models. Our results suggested that despite restricted replication of the vector in mice and hamsters, the live mucosal NDV-HXP-S conferred protection against viral replication and spread. Importantly, the mucosal multivalent vaccine retained the breadth of protection not only in naïve animals, but also in pre-immune animals.

## Materials and methods

### Production of NDV-HXP-S vaccine candidates

NDV-HXP-S vectors expressing the pre-fusion stabilized spike protein of ancestral, Beta and Delta variants were rescued and produced in specific pathogen free embryonated (SPF) chicken eggs as described in a previous work (3, 15, 16). Purified NDV-HXP-S vaccines were re-suspended in Phosphate Buffered Saline (PBS) (pH 7.4) and the infectious titer in 50% egg infectious doses (EID_50_) and the total protein concentration was measured as previously described (14).

### Rescue of the rNDV-luc virus

Mouse codon-optimized sequence of Firefly luciferase gene (GenBank ID: MN728548.1) was generated using the Integrated DNA Technologies (IDT) Codon Optimized Tool (IDT Technologies Inc, IA, USA). Luciferase sequence was synthesized using the gBlocks^®^ Gene Fragments service (IDT) and inserted into pNDV_LS/L289A rescue plasmid (between P and M genes) by in-Fusion cloning (Clontech) and rNDV virus was rescued and quantified as previously described (17).

### *In vitro* luciferase assay

BSR-T7 monolayers were infected with rNDV-luc at 0.5 multiplicity of infection. Cells were harvested 48 hours post infection and an *in vitro* luciferase kit (Rapid Detection of Firefly Luciferase Activity, Promega, WI, USA) was used to measure the luciferase activity in infected cells following the manufacturer’s instructions.

### Animal immunization experiments

All the animal experiments were performed in accordance with protocols approved by the Icahn School of Medicine at Mount Sinai (ISMMS) Institutional Animal Care and Use Committee (IACUC). All experiments with live SARS-CoV-2 were performed in the Centers for Disease Control and Prevention (CDC)/US Department of Agriculture (USDA)-approved biosafety level 3 (BSL-3) biocontainment facility of the Global Health and Emerging Pathogens Institute at the ISMMS, in accordance with institutional biosafety requirements.

### *In vivo* luciferase assay and bioluminescent live imaging

For mice biodistribution studies, 8-to-10-week-old female BALB/c mice (RRID: IMSR_JAX:000651, The Jackson Laboratory) were used for these experiments. An infectious dose of 10^5^ or 10^6^ EID_50_ of NDV was diluted in PBS (pH 7.4, Gibco Cat#10010-023) in a total volume of 30 μl for IM or IN administration (3). *In vivo* bioluminescence imaging was performed using a Biophotonic IVIS^©^‐Spectrum system (Perkinelmer, MA, USA) located at the BioMedical Engineering and Imaging Institute at ISMMS. Mice anesthetized by isofluorane were imaged 5 minutes after intraperitoneal injection with fresh D-Luciferin (150 mg/kg; Thermo Fisher Scientific). Different set of mice from one group were analyzed each day to allow them for a complete recovery from anesthesia. Luciferase signal was quantified using Living Image software (Caliper LifeSciences) in three different regions of interest (ROI) of each mouse.

### Live NDV-HXP-S vaccine biodistribution in Golden Syrian hamsters

Eight- to ten-week-old female Golden Syrian hamsters (HsdHan^®^:AURA, Inotiv) were vaccinated with 10^7^ EID_50_ of ancestral NDV-HXP-S vaccine or wildtype LaSota NDV (WT NDV) strains, as performed in previous work (3). Vaccines were diluted in PBS in a total volume of 20 µl or 50 µl per dose for its IN or IM administration (3), respectively. One- or seven-days post vaccination one subset of hamsters were euthanized, and several biological fluids including nasal washes, blood and urine and organs including lungs, brain, leg muscle at the site of the vaccination were collected. Nasal washes were collected in 0.4 mL of PBS with 100 µg/mL of P/S (pH 7.4, Gibco). Blood was retrieved using cardiac puncture and urine was extracted from the bladder. Nasal washes and urine were spun at 3,000 rpm for 20 min at 4°C and blood was spun at 5000 rpm for 30 min at 4°C and all samples were stored at -80 °C until further use. Right lung lobes were homogenized in 1 mL of PBS using a Lysing matrix A homogenization tubes (MP Biomedicals). Lung homogenate supernatant was collected after centrifugation (10,000 rpm x 10 min at room temperature, RT). Several organs including the left lung lobe, brain, leg muscle at the site of infection were collected and stored in 4% PFA (v/v) solution in PBS (pH 7.4, Gibco) overnight at RT and later embedded in paraffin as Formalin Fixed Paraffin Embedded tissue (FFPE) and stored at RT until use.

### Immunization and direct-contact transmission study in Golden Syrian hamsters

In the direct-contact transmission study, a subset of 8- to 10-week-old hamsters vaccinated at the same time as those in the biodistribution study were boosted five months later with a second dose of NDV-HXP-S IN or IM. The immunized animals were bled via gingival vein pre-boost and four-weeks after second boost. Thereafter, vaccinated hamsters (recipient) were placed in pairs with 8- to 10-week-old female Golden Syrian hamsters challenged with 10^5^ PFU of WA1-USA/2020 SARS-CoV-2 strain (donors) in a direct contact transmission setting. A healthy control group in which the hamsters were mock-challenged was included as control. Weight changes were monitored for 5 days. Nasal washes were collected at day 1 and day 3 post challenge for both donors and recipients, as previously described. Animals from each group were euthanized day 5 post-challenge to harvest nasal turbinate and lungs. Nasal turbinate, upper right lung lobe and lower right lung lobes were homogenized as previously described (21). Left lung lobe was stored in 4% PFA (v/v) and latter embedded in FFPE as previously described.

### Formalin-fixed Paraffin-Embedded RNA extraction

RNA extraction was performed using Maxwell RSC RNA FFPE Kit protocol following the manufacturer’s instructions (Catalog number AS1440, Promega) in the Biorepository and Pathology Core (ISMMS). A total of 50 µm scrolls (3 µm thickness each) were cut from FFPE block for RNA extraction. The isolated RNA was quantified using a Qubit RNA HS Assay Kit and Qubit 3.0 Fluorometer (<ns/>Cat Q32855, Thermo Fisher Scientific).

### NDV vRNA detection by RT-qPCR in hamster biological samples

A validated Reverse Transcriptase qPCR (RT-qPCR) method was used to quantify viral copies of the NP gene (**Table 1**) of WT NDV or NDV-HXP-S viruses in different tissues. Consensus sequences from the Golden Syrian hamster housekeeping genes β-actin (Gene ID: 101844587) and GAPDH (Gene ID: 121132788) were retrieved from NCBI and used as house-keeping controls. Primers were designed using SnapGene tool (**Table 1**) (18) and standards were generated in-house for each gene. Standards were 10-fold serially diluted in UltraPure™ DNase/RNase-Free Distilled Water (Thermo Fisher Scientific, MA, USA Cat<ns/> 10977015) resulting in 10^8^ to 10^1^ copies/sample. The efficiency, R^2^, slope and y-int used are present in **Table 1**. RT-qPCR was performed using the iTaq™ Universal SYBR Green One-Step Kit (BioRad, Hercules, CA) in the CFX Opus 96 Real-Time PCR Instrument 96-well (BioRad, Hercules, CA). A no-template control and a condition with 10^5^ copies of the amplicon in triplicate were added as internal negative and positive control, respectively. RT-qPCR was performed following the manufacturer thermal cycling conditions, except for the annealing and extension time which was modified to 20 sec at 55°C. Melt curve analysis was also performed to confirm amplification specificity. The copies per sample were finally interpolated from the different standard curves, then, the results were normalized to copies/ng.

**Table 1:** Primers and standard curve of the Golden Syrian hamster tissue RT-qPCR analysis.

Gene-specific primers	Sequence	Tm (°C)	GC%	Amplicon size
**NP FORWARD**	5’ – AGAGAGCACAGAGATTTGCG – 3’	57	50	128
**NP REVERSE**	5’ – GATCCTCTCCAGGGTATCGGT –3’	60.2	52.4	
**β-actin FORWARD**	5’ – GTGCTATGTTGCCCTGGACT – 3’	60	55	113
**β-actin REVERSE**	5’ – GCTCGTTGCCAATGGTGATG – 3’	59	55	
**GAPDH FORWARD**	5’ – CAAGTTCAAAGGCACAGTCA – 3’	54	50	152
**GAPDH REVERSE**	5’ – TGGTGGTGAAGACGCCAGTA – 3’	53	50	
Standard curve	Efficiency (%)	*R* ^2^	Slope	y-int
**NP**	95.6	0.996	-3.431	40.590
**β-actin**	102.2	0.994	-3.270	36.358
**GAPDH**	99.1	0.990	-3.345	37.380

### Intranasal Immunization of live NDV-HXP-S vaccines in naïve mice

Vaccinations were performed in female BALB/c mice (RRID: IMSR_JAX:000651, The Jackson Laboratory) using a two-dose regimen as indicated in each experiment. For IN vaccination, 8-to-10-week-old mice were anesthetized with a ketamine/xylazine cocktail before administration of 10 µl of total volume split between each nostril. For intramuscular (IM) vaccination, vaccines were prepared in 100 µl total volume and 50 µl were administered into the thigh muscle of each leg. The live concentrated NDV-HXP-S and its trivalent version were used at a total infectious dose of 10^6^ EID_50_, being a dose of 3.3x10^5^ EID_50_ for each variant vaccine when a trivalent formulation was used diluted in PBS (19).

### Mucosal samples collection

Mucosal samples were collected after mouse euthanasia. Mice nasal washes and bronchioalveolar lavage fluid (BALF) were collected in 1 mL total volume of PBS with 100 units (U)/mL of penicillin-streptomycin (P/S; Gibco Cat<ns/>15140122). Oral washes were collected in a total volume of 0.4 mL of PBS with 100 U/mL P/S (pH 7.4, Gibco). Vaginal lavages and intestinal lavages were collected in a total volume of 0.4 mL or 5 mL, respectively of PBS with 1% (v/v) anti-protease cocktail (Sigma Cat<ns/> P8340). Nasal washes, BALF, oral washes, intestinal lavages, and vaginal lavages samples were spun at 3000 rpm for 20 min at 4°C then the supernatant was collected and stored at -20°C until further use. Fecal homogenates were generated by diluting the feces to a concentration of 0.1 g/mL of PBS with 1% (v/v) anti-protease cocktail. Feces were then homogenized and centrifuged for 10 min at maximum speed (~14,000 rpm) at 4°C. The supernatant was then collected and stored at -20°C.

### Immunization with NDV-HXP-S vaccines in pre-immunized mice

129 mice (RRID: IMSR_JAX:002448, The Jackson 212 Laboratory) were used in the heterologous vaccine platform study (mRNA vaccination followed by NDV booster). 129 mice were vaccinated with Pfizer mRNA vaccine at 0.25 µg IM twice 3 weeks apart as performed in previous work (19, 20). Ten-months after the second dose, animals were boosted with NDV-based vaccines via IN or IM route as previously described (14, 19). Twenty-one days later animals were euthanized, and blood, spleen and mucosal samples were collected. The live concentrated NDV-HXP-S and its trivalent version were used at 10^6^ EID_50_ diluted in PBS. The inactivated monovalent and trivalent NDV-HXP-S vaccines were used at 1 µg of total protein diluted in PBS as previously described (14).

### Immunization and viral challenge study in Golden Syrian hamsters

Naïve 8- to 10-week-old female Golden Syrian hamsters were vaccinated with a two-dose vaccination regimen at 10^6^ EID_50_/animal of ancestral NDV-HXP-S, trivalent NDV-HXP-S vaccines, or wild type LaSota NDV (WT NDV) strain in a 12-week interval (21). Vaccines were diluted in PBS (Gibco) in a total volume of 20 µl or 100 µl per dose for its IN or IM administration, respectively. Hamsters were bleed 12-weeks post boost and challenged with 5x10^4^ PFU of Omicron BA.1 SARS-CoV-2 strain. Weight changes were monitored for 5 days. Throat swabs were collected and viruses were lysed in 0.3 mL Qiagen lysis buffer at days 1, 3 and 5 post challenge and stored at -80 °C until further processing. Animals from each group were euthanized day 5 post-challenge to harvest nasal turbinate and lungs. Nasal turbinate, upper right lung lobe and lower right lung lobes were homogenized as previously described (21).

### Histopathology and immunohistochemistry

FFPE left lung tissues obtained from hamsters were cut into 5 μm sections and stained by the Biorepository and Pathology Core (ISMMS). The presence of NDV viral antigens was detected with a rabbit polyclonal serum (1:2000 dilution, brown) generated by Covance (labcorp, Princeton, NJ, USA) through immunization rabbit with adjuvanted inactivated NDV viruses and a human mAb 1A9 (Cat<ns/> MA5-35946, Thermofisher) anti SARS-CoV-2 spike antibody (1:2000 dilution, pink). SARS-CoV-2 nucleoprotein (N) was stained using a mouse anti-N monoclonal antibody 1C7C7 (1:50 dilution, brown) kindly provided by Dr. James Duty at ISMMS (Millipore Sigma, cat. no. ZMS1075) (3, 21). Briefly, IHC staining was performed using VENTANA DISCOVERY ULTRA automated slide staining instrument (Roche, Basilea, Switzerland). Single staining (automatic or semi-automatic) was performed using the selected primary antibody and the and Discovery OMNIMap anti-host-HRP (Roche) as secondary antibody. The signal was obtained using Discovery ChromoMap DAB RUO (760–2513) (depicted in brown signal). For double staining, the same procedure was performed sequentially. Briefly, after the first staining (depicted in brown), slides went throughout a step of inhibition, heat denaturation and neutralization and then the second primary antibody was applied followed a secondary and a developing kit (depicted in pink). Tissues were counterstained with Hematoxylin to visualize the nuclei. Whole tissue sections on the slide will be converted into high-resolution digital data using a NanoZoomer S210 Digital slide scanner (Hamamatsu). The HALO image analysis platform was used for quantitative tissue analysis (Indica Labs, Inc.), using random forest algorithm classifier. Multiplex IHC module and color deconvolution were used to separate chromogenic stains together with nuclei segmentation to set up the system for quantitative analysis.

### SARS-CoV-2 RT-qPCR

RNA was extracted from throat swab samples using the QIAamp Viral RNA Mini Kit (QIAGEN, Germantown, MD) according to the manufacturer’s instructions. Genomic copies of SARS-CoV-2 Nucleocapsid (N) gene were measured as previously described (22). Briefly, Forward Primer 5’-GACCCCAAAATCAGCGAAAT-3’ and Reverse Primer 5’-TCTGGTTACTGCCAGTTGAATCTG-3’ were used, yielding a 72 bp amplicon. Standard curves were generated using the 2019-nCoV_N_Positive Control plasmid (IDT, Cat No.10006625), which encodes the N gene of SARS-CoV-2 isolate Wuhan-Hu-1 (GenBank: NC_045512.2) (**Table 2**). RT-qPCR was performed using the iTaq™ Universal SYBR Green One-Step Kit and the CFX Opus 96 Real-Time PCR System following the manufacturer recommended thermal cycling conditions (39 cycles of 30 sec at 55°C of anneal and extension). Copies per sample were afterwards calculated (obtained copies/5 µl of RNA extracted per reaction x 60 µl of total RNA extracted per sample).

**Table 2:** Standard curve of the SARS-CoV-2 N RT-qPCR analysis.

Standard curve	Efficiency (%)	R^2^	Slope	y-int
**N**	108.5	0.993	-3.133	39.558

### Enzyme-linked immunosorbent assay

Spike-specific IgG and IgA antibody titers in mice and hamster samples were measured by ELISA as described previously (14, 16, 23). Proteins were coated onto Immulon^®^ 4 HBX 96-well microtiter plates (Thermo Fisher Scientific, Cat<ns/>3855) at 2 μg/mL in 1x coating buffer (SeraCare Life Sciences Inc. USA Cat<ns/>5150-0014 (50–84–00), MA, USA) at 50 μL/well overnight at 4°C. All plates were washed 3 times with 225 μL PBS containing 0.1% (vol/vol) Tween-20 (PBST) and 220 μL blocking solution (3% goat serum, 0.5% non-fat dried milk powder, 96.5% PBST) was added to each well and incubated for 1 hour at RT. Samples were serially diluted in blocking solution followed by a 2-hour incubation at RT at a starting dilution of 1:30 for serum samples and 1:2 dilution for mucosal samples. ELISA plates were afterwards washed 3 times with PBST and 50 μL of anti- IgG (1:3,000) or anti -IgA (1:2,000)-horseradish peroxidase (HRP) conjugated antibody (Cytiva, GE Healthcare) was diluted in blocking solution. After 1 hour, plates were washed 3 times with PBST and developed using SigmaFast OPD (Sigma-Aldrich Cat<ns/> P9187, MI, USA) for 10 minutes. Reactions were stopped by adding 50 μL 3M hydrochloric acid and absorbance at 492 nm was determined on a Synergy 4 plate reader (BioTek, Agilent Technologies inc., CA, USA) or similar. For each ELISA plate, the blank average absorbance plus 3 standard deviations were used as a cutoff to determine endpoint titers and the area under the curve (AUC) using GraphPad Prism.

### Microneutralization assays using the authentic SARS-CoV-2 viruses

Microneutralization assays using the authentic SARS-CoV-2 viruses were performed as described previously in Vero E6-TMPRSS2 (24). Vero-TMPRSS2 cells were seeded in 96-well high binding cell culture plates (Costar Cat<ns/> 07620009, Corning) at a density of 20,000 cells/well in complete Dulbecco’s modified Eagle medium (cDMEM Cat<ns/>10-013-CV, Corning) one day prior to the infection. Heat-inactivated serum samples (56°C for 1 hour) were serially diluted (3-fold) in minimum essential media (MEM Cat<ns/>11430-030, Gibco) supplemented with 2 mM L-glutamine (Gibco Cat<ns/>25030081), 0.1% sodium bicarbonate (w/v, HyClone Cat<ns/>SH30033.01), 10 mM 10 mM 4-(2-hydroxyethyl)-1-piperazineethanesulfonic acid (HEPES, Cat<ns/> 15630080 Gibco), 100 U/ml penicillin, 100 μg/ml streptomycin (P/S; Gibco) and 0.2% BSA (MP Biomedicals Cat<ns/>810063, CA, USA) starting at 1:10. Remdesivir (Medkoo Bioscience inc. Cat<ns/> 329511, NC, USA) was included to monitor assay variation. Serially diluted sera were incubated with 10,000 TCID_50_ per mL of Wuhan-like ancestral USA-WA1/2020 and PV44488/2021 (B.1.1.529, Omicron) for one hour at RT, followed by the transfer of 120 μl of the virus-sera mix to Vero E6-TMPRSS2 plates. Infection proceeded for one hour at 37°C and inoculum was removed. One hundred μl/well of the corresponding antibody dilutions plus 100 μl/well of infection media supplemented with 2% Fetal Bovine Serum (FBS, Cat<ns/>10423-028, Gibco) were added to the cells. Plates were incubated for 48 h at 37°C followed by fixation overnight at 4°C in 200 μl/well of a 10% formaldehyde solution. For staining of the nucleoprotein, formaldehyde solution was removed, and cells were washed with PBS (pH 7.4 Gibco) and permeabilized by adding 150 μl/well of PBS, 0.1% Triton X-100 (Fisher Bioreagents Cat<ns/> BP151-100, MA, USA) for 15 min at RT. Permeabilization solution was removed, plates were washed with 200 μl/well of PBS (Gibco) twice and blocked with PBS, 3% BSA for 1 hour at RT. During this time the primary antibody was biotinylated according to manufacturer protocol (Thermo Scientific EZ-Link NHS-PEG4-Biotin). Blocking solution was removed and 100 μl/well of biotinylated mAb 1C7C7 at a concentration of 1μg/ml in PBS, 1% BSA was added for 1 hour at RT. Cells were washed with 200 μl/well of PBS twice and 100 μl/well of HRP-conjugated streptavidin (Thermo Fisher Scientific) diluted in PBS, 1% BSA were added at a 1:2,000 dilution for 1 hour at RT. Cells were washed twice with PBS, and 100 μl/well of SigmaFast OPD (Sigma-Aldrich) were added for 10 min at RT, followed by addition of 50 μl/well of a 3 M HCl solution (Thermo Fisher Scientific). Optical density (OD) was measured (490 nm) using a microplate reader (Synergy H1; Biotek). Analysis was performed using GraphPad Prism 7 software. After subtraction of background and calculation of the percentage of neutralization with respect to the “virus only” control, a nonlinear regression curve fit analysis was performed to calculate the 50% inhibitory dilution (ID_50_), with top and bottom constraints set to 100% and 0% respectively. All samples were analyzed in a blinded manner.

### SARS-CoV-2 plaque assay

The plaque assay was performed in the BSL-3 facility of the Icahn School of Medicine at Mount Sinai. The day before the assay, 3 x 10^5^ cells of Vero-E6 cells or Vero-E6 TMPRSS2 T2A ACE2 cells were seeded in 12-well plates (Thermo Fisher Scientific) as previously described (3, 19). Lung and nasal turbinate homogenates were 10-fold serially diluted in infection medium (DMEM + 2% FBS + 1% P/S + 10 mM HEPES). After removing the media from the cells, 200 μL of each tissue homogenate dilution were inoculated onto each well. The dilutions used range from 10^-1^ to 10^-6^. The plates were incubated at 37°C for 1h with rocking every 15 min. Subsequently, the inoculum was removed and 1 mL of agar overlay consisting of 0.7% agar in 2x MEM + 2% FBS was placed onto each well. Once the agar was solidified, the plates were incubated at 37°C with 5% CO_2_. Two days later, the plates were fixed with 4% formaldehyde in PBS overnight before being taken out of the BSL-3 facility for subsequent staining in BSL-2 facility. The plaques were immuno-stained with an anti-SARS-CoV-2 NP primary mouse monoclonal antibody 1C7C7 kindly provided by Dr. James Duty at ISMMS (3, 21). Amersham ECL sheep anti-mouse IgG Horseradish Peroxidase (HRP)-linked whole secondary antibody (Cytiva Cat<ns/> NA931, RRID: AB_772210) was used at 1:2,000 and the plaques were visualized using TrueBlue Peroxidase Substrate (SeraCare Life Sciences Inc., <ns/>5510-0030).

### Intracellular cytokine staining

Splenocyte isolation and intracellular cytokine staining was performed adapted from (25). Briefly, single suspension splenocytes were resuspended in R10 media and seeded in U-bottom 96-well plates (CELLSTAR, Greiner Bio-One North America Inc., Monroe, NC, USA) at an average of 2 ×10^6^ cells/well containing anti-mouse CD28 (1:500, BD Biosciences Cat<ns/> 557393), brefeldin A (1:1000, GolgiPlug™, BD Biosciences Cat<ns/> 555029), and monensin (1:1,000, GolgiStop™, BD Biosciences Cat<ns/> 554724). Splenocytes were stimulated with PepMix™ SARS-CoV-2 or PepMixTM SARS-Cov-2 (Spike B.1.1.529/BA.1/Omicron, Cat<ns/>PM-WCPV-2, Cat<ns/>PM-SARS2-SMUT08-1, JPT Peptides), at a final individual peptide concentration of 5 µg/mL at 37 °C with 5% CO_2_ for 8-10 hours. Negative control cells were stimulated with an equivalent volume of DMSO. Positive control cells were stimulated with a cocktail containing phorbol 12-myristate 13-acetate (PMA, 0.5 mg/mL, Sigma-Aldrich) and ionomycin (1 mg/mL, Sigma-Aldrich). The unstimulated control cells were only treated with the R10 media. After stimulation, cells were washed with PBS containing 2% FBS and centrifuged at 350 x g for 5 min and then stained with Zombie Red™ diluted in PBS (1:500, BioLegend Cat<ns/> 423109) for 15 min at RT in the dark. Cells were washed in PBS containing 2% FBS (350 x g for 5 min) and incubated with surface staining cocktail containing Fc Block CD16/CD32 1:50 (BD Biosciences Cat<ns/> 553141, RRID: AB_394656) and the anti-mouse antibodies BV 711 CD3 (1:300, clone 17A2, BD, Cat<ns/> 100241, RRID: AB_256394), Pacific Blue CD4 (1:400, clone GK1.5, BioLegend, Cat<ns/> 100428, RRID: AB_493647), PerCP/Cy5.5 CD8 (1:200 clone 53-6.7, BioLegend, Cat<ns/>100734, RRID: AB_2075238) for 30 min at 4 °C in FACS buffer. Cells were washed in FACS buffer and then incubated in fixation/permeabilization buffer (CytoFix, BD Biosciences) for 5 min at 4°C. After fixation, cells were washed in 1 x permeabilization buffer (CytoPerm, BD Biosciences), then incubated with the intracellular staining cocktail containing anti-mouse antibodies Alexa Fluor 647 IFN-γ (1:400, clone XMG1.2, BioLegend, Cat<ns/> 505814, RRID: AB_49331), Alexa Fluor 488 TNF-α (1:300, clone MP6-XT22, BioLegend, Cat<ns/> 506313, RRID: AB_493328), PE/Cy7 IL-2 (1:300, clone JES6-5H4, BioLegend, Cat<ns/> 503832, RRID: AB_2561750), in 1x permeabilization buffer for 1 h at 4 °C. Samples were then washed in 1x permeabilization buffer and resuspended in PBS buffer for acquisition. Samples were acquired on an Aurora spectral cytometer (Cytek, Fremont, CA, USA) using SpectroFlo^®^ software (Cytek). Analysis was performed with FCS Express 7 (DeNovo Software) and GraphPad Prism 9.5.1 (GraphPad Software 10.4.0).

### Blood SARS-CoV-2 S-specific T cell surface staining

One volume of blood was collected into four volumes of 0.5 M EDTA in centrifuge tubes to prevent coagulation, then pooled by group as previously described (19). Whole blood was stained with the direct addition of 1 μL of PE-conjugated H-2K(b) VNFNFNGL Tetramer (1:100, NIH Tetramer Core Facility). Tubes were flicked to mix reagents then incubated for 40 min at RT in the dark. After incubation, the following antibodies or fluorescent dyes were added directly to tubes containing tetramer-stained whole blood: 1 μL of Fc Block (BD, clone 2.4G2, RRID: AB_394656), 1 μL of CD8α PerCP (clone 53-6.7, BD, RRID: AB_394573), 1 μL of CD3ϵ Alexa Fluor 700 (clone 17A2, BioLegend, RRID: AB_493696), 0.5 μL of MHC II eFluor 450 (clone M5/115.15.2, Invitrogen, RRID: AB_1272204), and 0.5 μL of Fixable Viability Dye eFluor 450 (Invitrogen). Tubes were flicked to mix and incubated at RT for 20 min in the dark. After incubation, 1 mL of staining buffer was added to each tube to quench staining, then tubes were centrifuged at 400 x g for 5 min at 4°C. Supernatants were removed using a pipette, then to fix and lyse RBCs, cell pellets were resuspended with 200 μL of 1x eBioscience Foxp3/Transcription Factor Fixation/Permeabilization Buffer (Invitrogen), prepared according to manufacturer instructions. Cells were incubated for 10 min at RT in the dark. After incubation, cells were washed twice by adding 1 mL of staining buffer to each tube, centrifuging at 400 x g for 5 min at 4°C, then removing supernatants. After the final wash, cell pellets were resuspended with 200 μL of staining buffer. To prepare compensation controls, 1μL of each antibody was added to 1 drop of UltraComp eBeads Plus (Invitrogen) and incubated for 15 minutes before 200μL of staining buffer was added to the tube. Stained blood samples were measured on a Beckman Coulter Gallios flow cytometer equipped with Kaluza data acquisition software. Analysis was performed using FlowJo 10.8.1 (Treestar) and compensated using the built-in AutoSpill algorithm.

### Statistics

Paired t-test, one-way ANOVA, two-way-ANOVA or non-parametric Kruskal Wallis test were used to compare the different experiments. Statistical analyses were performed using Prism software (GraphPad 10.4.0).

## Results

### Biodistribution of Newcastle disease virus in mice and hamsters

**Figure 1:**
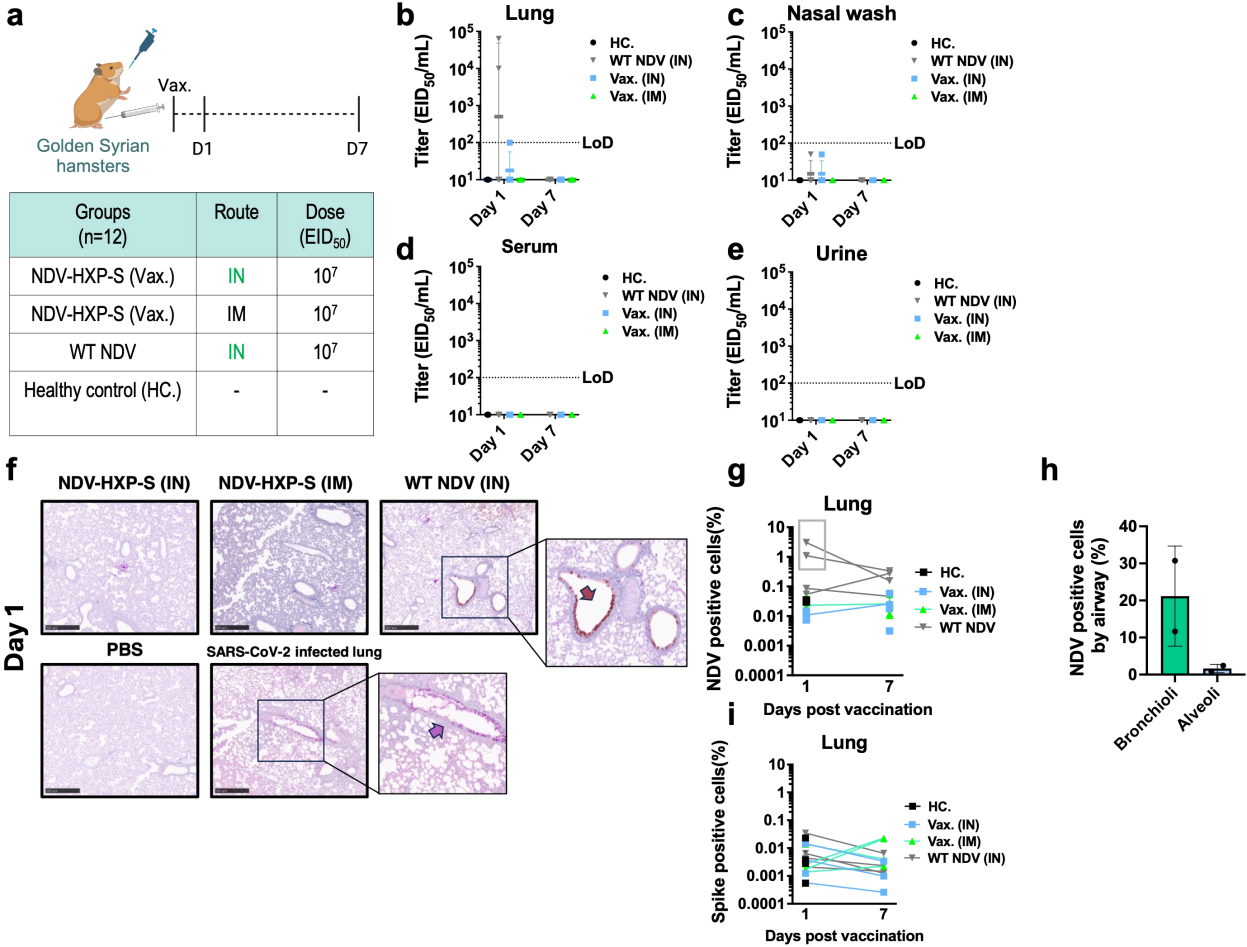
Biodistribution of live NDV-HXP-S vaccine in Golden Syrian hamsters. **(a)** Experimental design and vaccination groups. Golden Syrian hamsters (n=12) were immunized with a total dose of 10^7^ EID_50_ of ancestral NDV-HXP-S via IN or IM route. Two more groups vaccinated with NDV LaSota (WT) via IN and PBS were used as controls. A total of 4 hamsters were analyzed per time point **(b–e)** At day 1 and day 7 after the vaccination, **(b)** lung homogenates, **(c)** nasal wash, **(d)** blood serum and **(e)** urine were collected and presence of infectious NDV was checked by injecting 100 µL of each biological fluid into each of the specific pathogen-free (SPF) embryonated chicken eggs. Viral titers were subsequently measured by EID_50_. (limit of detection equals to 100 EID_50_/mL; a titer of 10 EID_50_/mL was assigned to HA negative samples, and a titer of 50 EID_50_/mL was assigned to HA positive samples with no EID_50_/mL titer). **(f-i)** Left lobe was fixed in 4% paraformaldehyde in PBS (4% PFA, v/v) overnight and analyzed by immunohistochemistry (IHC) at day 1 and day 7 post vaccination. Presence of NDV viral protein or spike expression was measured with a rabbit polyclonal anti-NDV serum 1:2000 (shown in brown) or a human anti-SARS-CoV-2 spike 1A9 antibody 1:2000 (shown in pink). Examples of positively stained regions are indicated with arrows. Golden Syrian hamster lungs infected with SARS-CoV-2 (USA-WA1/2020), were used as spike staining positive control. The percentage of **(g)** NDV and **(i)** spike positive cells in each slide and **(h)** the percentage of NDV positive cells by airway in the two lung samples from the 1-day post administration of WT NDV group were measured with HALO software.

A bioluminescence-based live imaging assay was first used to investigate the NDV tissue distribution in the mouse model. A recombinant NDV vector expressing an intracellular Firefly luciferase was used as a reporter virus for *in vivo* imaging (rNDV-luc, **Supplementary Figure S1A**) (17). The activity of the luciferase expressed by the rNDV-luc was confirmed in BSR-T7 cells upon infection (**Supplementary Figure S1B**) and in mice that were intranasally infected with rNDV-luc (FigureS1C-D). For live-imaging biodistribution study, rNDV-luc was administered intranasally (IN) or intramuscularly (IM) at 10^6^ EID_50_ or 10^5^ EID_50_ into BALB/c mice. Bioluminescence was measured every day until the signal was no longer detected. Luciferase activity peaked at 24 hours primarily at the site of administration and quickly declined to baseline after three days (**Supplementary Figure S1E-I**). We observed a very high luminescent signal in the lungs of mice that received rNDV-luc IN at 24 hours post inoculation (**Supplementary Figure S1E, G**). A transient increase of signal, that was much lower than that in the lungs, was observed in the abdomen of mice that received rNDV-luc IN, likely due to the swallowing of the material (**Supplementary Figure S1H**). In addition, we observed a slight increase of luminescence signal in the legs of mice that received the high-dose vaccine IM - also at 24 h post inoculation (**Supplementary Figure S1E, I**), although the difference was not significant. Overall, NDV was observed to be restricted in replication in mice (26–28).

Next, we investigated biodistribution of the live NDV-HXP-S vaccines. The prototype ancestral NDV-HXP-S was chosen as a representative since similar replication kinetics have been reported for all NDV-HXP-S variant vaccines (14, 21).Golden Syrian hamsters were selected due to their natural expression of the ACE2 receptor and their natural susceptibility to SARS-CoV-2 infection (29, 30). Hence, NDV-HXP-S virus tropism compared to the wildtype vector could be evaluated. The ancestral NDV-HXP-S vaccine was administered IN or IM at a dose of 10^7^ EID_50_. The presence of NDV and the expression of the spike protein was evaluated after 1 day and 7 days post vaccination. A group vaccinated with the wild type NDV LaSota (WT NDV) and an unvaccinated group were included as controls (**Figure 1a**). The presence of infectious NDV was evaluated in lung homogenates, nasal washes, blood serum and urine samples by injecting 100 µl of the biological fluid into specific-pathogen free (SPF) embryonated chicken eggs. If infectious NDV is present, it is expected to present hemagglutination (HA) activity after incubation, which can be measured by HA assay. Then, the titer could be quantified by EID_50_ titrations as previously described (3) (**Figures1b-e**). Among all the biological samples collected, infectious NDV was only detected in two out of four lung homogenate samples of the WT NDV IN group and in one out of four lung homogenate samples from the NDV-HXP-S IN group, one day post-administration (**Figure 1b**). Importantly, these titers were much lower than the dose administered, suggesting a restricted replication of both WT NDV and NDV-HXP-S vaccine in the hamster model. Of note, NDV-HXP-S appeared to be attenuated as compared to the WT NDV based on the viral titers in the lung homogenates.

To examine the tropism of NDV-HXP-S relative to WT NDV in the respiratory tract, double staining of spike and NDV antigens by immunohistochemistry (IHC) was performed on formaldehyde-fixed parafilm embedded (FFPE) lungs on day 1 and day 7 after vaccination (**Figure 1f**). The presence of NDV infected cells was only detected in two samples in the WT NDV group on day 1 post-administration (highlighted within the grey rectangle, **Figures 1f, g**). In the WT NDV positive samples, bronchioles presented a higher degree of infection than the alveoli (**Figure 1h**). No presence of spike expression was detected in any of the samples (**Figure 1i**). In addition, viral genomic copies of NDV were quantified by reverse transcription-quantitative PCR in different tissues (RT-qPCR, **Supplementary Figure S2**). In agreement with replicative NDV shedding, high levels of viral RNA were only detected in the two lung homogenate samples from the WT NDV group that had measurable titers of infectious NDV (**Supplementary Figure S2A**). No viral RNA was detected in the brain (**Supplementary Figure S2D**). Very low levels of RNA were detected in leg muscles of animals that received the NDV-HXP-S IM (**Supplementary Figure S2G**).

### Protection of NDV-HXP-S vaccine against SARS-CoV-2 transmission to hamsters via contact with infected animals

**Figure 2:**
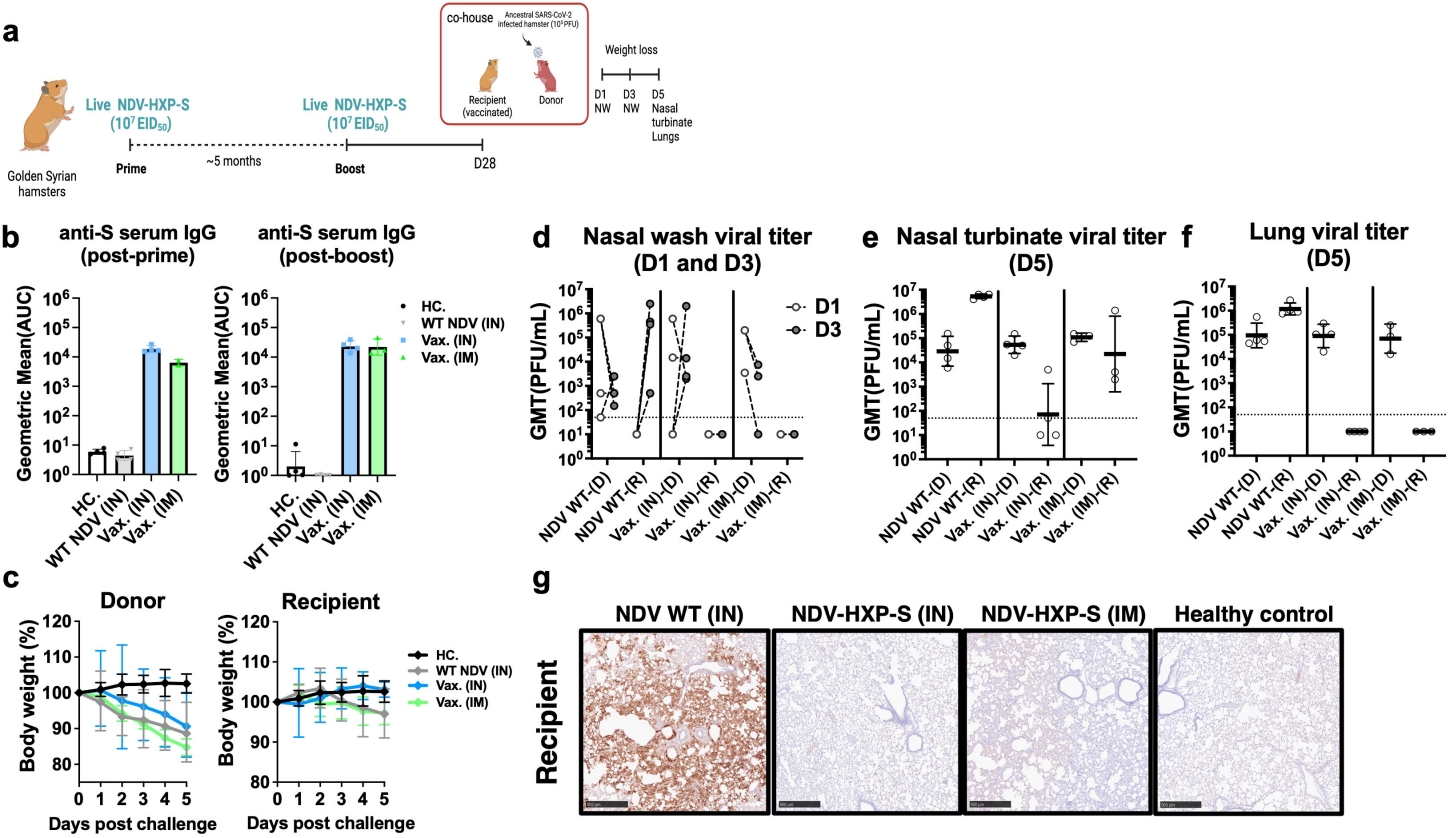
Live intranasal NDV-HXP-S vaccination reduces SARS-CoV-2 viral shedding in the upper respiratory tract in direct-contact transmission study. **(a)** Experimental design. Eight- to ten-week-old female Golden Syrian hamsters (n= 3 or 4) were either vaccinated with 10^7^ EID_50_ of live NDV-HXP-S intranasally (IN) or intramuscularly (IM), or WT NDV IN (negative control). Two immunizations were performed with 5-month interval. Twenty-eight days after second boost, naïve hamsters were challenged with 10^5^ PFU of ancestral (USA-WA1/2020) strain (Donors) and co-housed in pairs with vaccinated hamsters from different vaccinated groups (Recipient) and transmission was monitored in a direct-contact model. **(b)** Spike-specific IgG serum antibody titers were measured 5 months post-prime and 28 days post-boost, respectively. The geometric mean titer (GMT) of the area under the curve (AUC) ± the standard deviation (SD) is depicted. **(c)** Weight loss was monitored for 5 days. **(d)** Nasal washes were collected 1 and 3 days post challenge. At 5 days post challenge, **(e)** nasal turbinate and **(f)** right lung lobes were harvested and homogenized in 1 mL PBS and SARS-CoV-2 titer was measured by plaque assay. (D: Donor, R: recipient) **(g)** Left lobes were fixed in 4% PFA (v/v) and analyzed by IHC with a mouse anti-N monoclonal (1C7C7) (1:50, brown). Viral titers were measured by plaque assay on Vero E6 cells and were plotted as GMT of PFU/mL (limit of detection equals to 50 PFU/mL; a titer of 25 PFU/mL was assigned to negative samples).

A direct-contact transmission study was set up to test whether IN administration of NDV-HXP-S would enhance mucosal immunity and reduce viral shedding and transmission (31). To do so, a subset of Golden Syrian hamsters that were previously vaccinated with 10^7^ EID_50_ of the live NDV-HXP-S (IM or IN, **Figure 1a**) were boosted again with the same regimen (**Figure 2a**). A robust humoral immune response was observed in serum samples even only after 1 dose (**Figure 2b**) in agreement with previous data (3, 21). Four weeks after the second boost, naïve Golden Syrian hamsters (Donors) were challenged with ancestral-like virus (USA-WA1/2020) at a dose of 10^5^ plaque forming units (PFU) and co-housed with previously vaccinated hamsters (Recipients) (**Figure 2a**). Infected naïve donors showed weight loss after challenge (**Figure 2c**). In recipient hamsters, weight loss was only observed in WT NDV (grey line) or vaccinated IM groups (green line). But no weight loss was observed in hamsters vaccinated IN. (**Figure 2c**). SARS-CoV-2 shedding was measured in nasal washes on day 1 and day 3 post challenge (**Figure 2d**). Viral titers were detected in all donors (D) as well as WT NDV recipients (R), but not in the vaccinated ones. Viral titers at day 5 post-challenge measured in the nasal turbinate showed the greatest reduction in IN NDV-HXP-S vaccinated hamsters compared to the control group (WT NDV) (**Figure 2e**). Lower respiratory tract was equally protected by both IN and IM vaccinations, showing no detectable infectious SARS-CoV-2 in the lungs (**Figure 2f**) or SARS-CoV-2 antigens as measured by IHC (**Figure 2g**). This study demonstrated that the same live NDV-HXP-S vaccine that was administered via the mucosal route is better at preventing viral transmission than it administered systemically.

### Immunogenicity of an intranasal trivalent NDV-HXP-S vaccine in mice

In previous studies, we described the development of novel NDV-HXP-S variant-based vaccines (14, 19) and the possibility to combine them in a multivalent formulation to extend protection to mismatched strains not present in the formulation (14). Here, we further investigated the use of this prototype multivalent NDV-HXP-S formulation as a mucosal vaccine. This vaccine combines ancestral (Wuhan), Beta and Delta NDV-HXP-S in equal amounts (14). BALB/c mice were vaccinated in a 2-dose regimen with live trivalent NDV-HXP-S vaccine and spike-specific IgA titers were examined at different mucosal surfaces (**Supplementary Figure S3**) (14, 32, 33). The live trivalent vaccine induced mucosal spike-IgA at local mucosae (nasal wash, BAL, mouthwash) (**Supplementary Figure S3C-E**), but also at distal mucosae (vaginal lavage, intestinal lavage and feces samples) (**Supplementary Figure S3F-H**), demonstrating that the live NDV vector is a strong stimulator of mucosal immunity (34).

**Figure 3:**
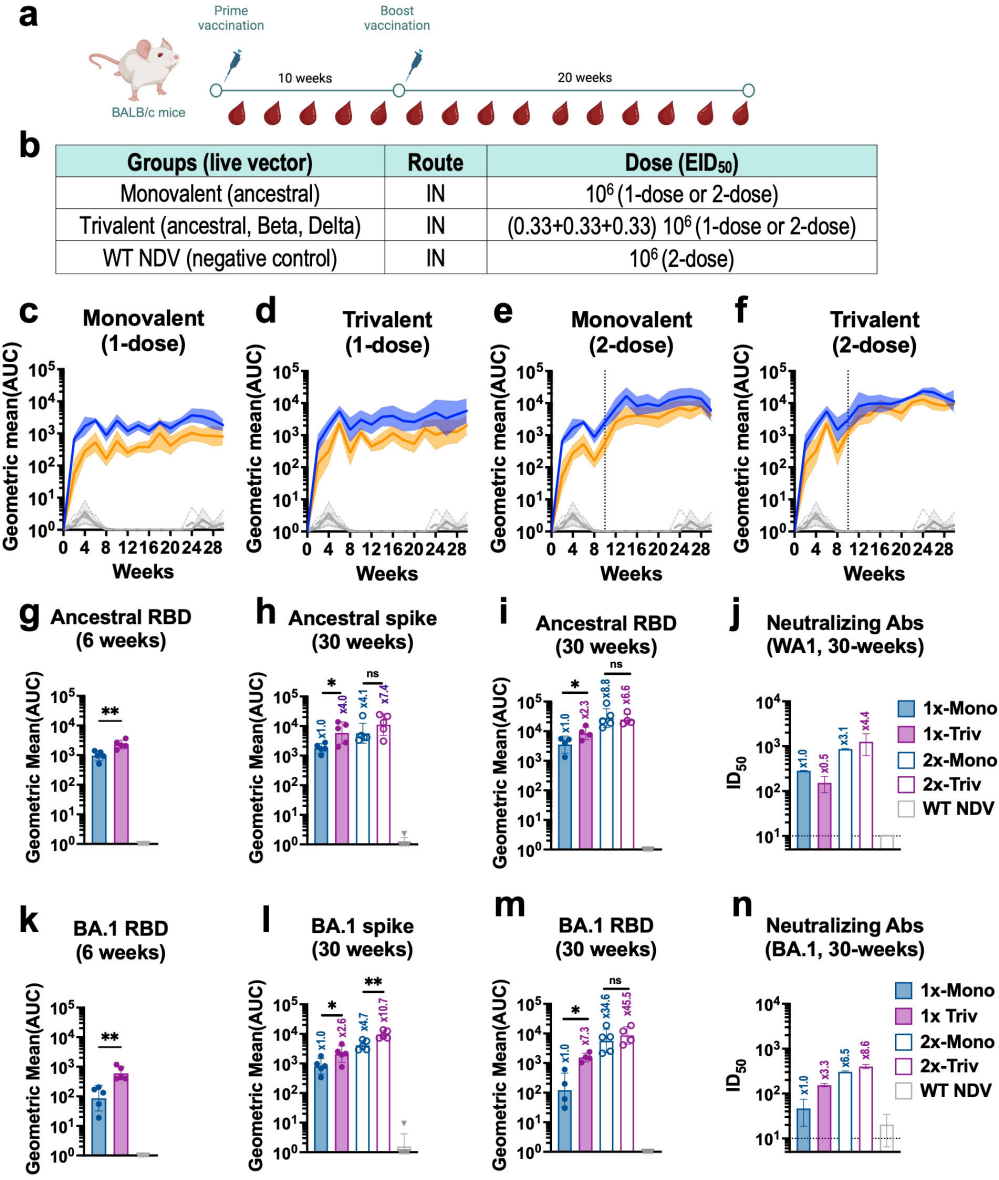
Live intranasal trivalent NDV-HXP-S vaccination induces superior cross-reactive humoral immune responses against phylogenetically distant SARS-CoV-2 variants in mice. **(a)** Experimental design and **(b)** vaccination groups. Eight- to ten-week-old female BALB/c mice were vaccinated with 10^6^ EID_50_ of live NDV-HXP-S monovalent or trivalent vaccines administered intranasally (IN) in a 1- or 2-dose regimen with a ten-week interval between doses. Mice vaccinated with WT NDV at a dose of 10^6^ EID_50_ were used as negative control. **(c-f)** Spike-specific IgG serum antibody titer kinetic (n=5) against ancestral (Wuhan, blue) or Omicron BA.1(B.1.1.529, orange) spike was measured every other week by ELISA for thirty -weeks. Individual kinetics for **(c)** monovalent 1-dose, or **(e)** 2-doses, **(d)** trivalent 1-dose or **(F)** 2-doses, are shown respectively. Spike-specific serum IgG of NDV WT vaccinated group against ancestral (light grey dotted line) or Omicron BA.1 (dark grey line) are also depicted in these graphs for comparison. Ancestral serum IgG antibody titers against **(g)** RBD at 6-weeks, **(h)** Spike at 30-weeks, **(i)** RBD at 30-weeks and **(j)** microneutralization assays against ancestral virus (USA-WA1/2020) at 30-weeks. Omicron BA.1 serum IgG antibody titers against **(k)** RBD at 6-weeks, **(l)** Spike at 30-weeks, **(m)** RBD at 30-weeks and **(n)** microneutralization assays against Omicron BA.1. One-tailed unpaired t-test are depicted (*p < 0.05; **p < 0.01; ***p < 0.001; ****p < 0.0001). Microneutralization assays against ancestral (USA-WA1/2020) **(j)** and Omicron BA.1 (n) were performed in technical duplicated from pooled sera. GMT ± SD is depicted.

We then investigated the kinetics of humoral immune responses induced by IN vaccination in mice. BALB/c mice were vaccinated with live monovalent or trivalent NDV-HXP-S at 10^6^ EID_50_ in a 1-dose or 2-dose regimen (**Figures 3a, b**). Spike-specific IgG antibody titers against ancestral Wuhan and Omicron BA.1 spike proteins (as a surrogate of cross-reactivity) were measured every other week for 30 weeks (**Figures 3c–f**). A dose-dependent and formulation-dependent kinetics was observed. Spike-specific IgG serum antibody titers presented an exponential increase in the first 6 weeks that later plateaued (**Figures 3c, d**). In the case of the 2-dose regimen, antibody titers were boosted by the second dose given at week-10 (**Figures 3e, f**). At the early time point of week-6, 1-dose of trivalent vaccine already presented higher antibody titers than 1-dose of monovalent vaccine (**Figures 3g, k**). The high spike and RBD antibody titers against both Wuhan (**Figures 3h, i**) and Omicron BA.1 (**Figures 3l, 3m**) showed by 2-dose trivalent NDV-HXP-S correlated with high neutralizing capacity against both viruses (**Figures 3j, n**), confirming the great potential of this new mucosal formulation.

### Protection of a trivalent vaccine against BA.1 challenge in hamsters

**Figure 4:**
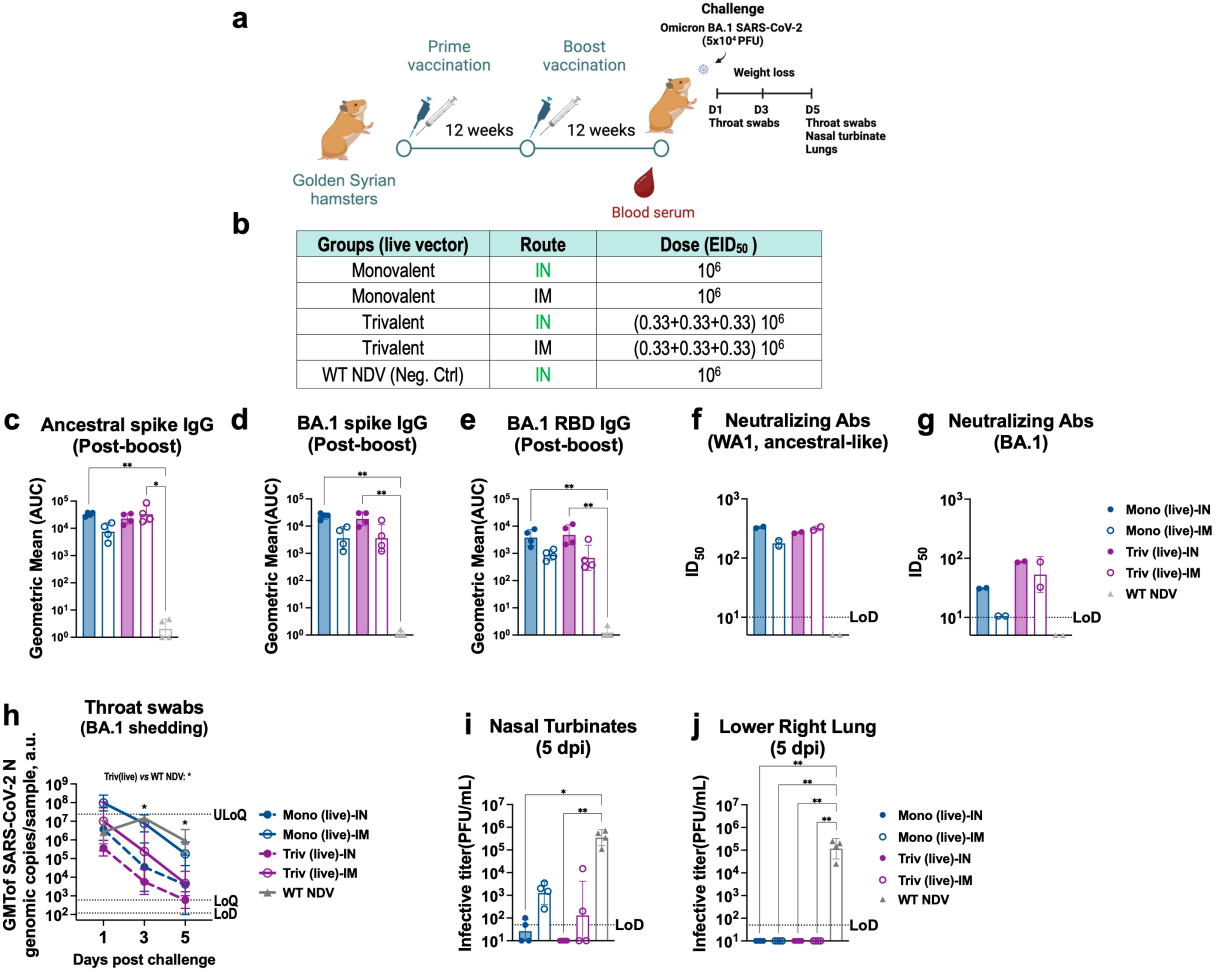
Intranasal and intramuscular vaccination of live trivalent NDV-HXP-S confer better protections against BA.1 than the monovalent vaccines in hamsters. **(a)** Experimental design and **(b)** groups. Eight- to ten-week-old female Golden Syrian hamsters (n= 4) were either vaccinated with live monovalent (Mono) or trivalent (Triv) NDV-HXP-S or WT NDV (negative control), intranasally (IN, green) or intramuscularly (IM, blue). Two immunizations were performed with 12-week interval. Twelve weeks after the second boost, hamsters were challenged with 5x104 PFU of BA.1 (B.1.1.529) strain and infection was monitored for five days. **(c)** Ancestral Spike-specific **(d)** Omicron BA.1 Spike-specific or **(e)** RBD-specific serum IgG antibody titers, neutralizing antibodies against **(f)** ancestral-like and **(g)** Omicron BA.1strains were measured 12 weeks post-boost vaccinations, respectively. The geometric mean titer (GMT) of the area under the curve (AUC) ± the standard deviation (SD) is depicted. **(h)** Throat swabs were collected 1, 3 and 5 days post-challenge and SARS-CoV-2 N genomic copies per sample were measured by RT-qPCR (limit of detection [LoD] equals to 120 copies; limits of quantification [LoQ] equals 600 copies and upper limit of quantification [ULoQ] equals to 2.4 x107 copies). **(i)** Nasal turbinate and **(j)** lower right lung lobes were harvested and homogenized in 1 mL PBS. Viral titers were measured by plaque assay on Vero E6-TMPRSS2-ACE2 cells and were plotted as GMT of PFU/mL (limit of detection [LoD] equals to 50 PFU/mL; a titer of 10 PFU/mL was assigned to negative samples). Non-parametric Kluskal-Wallis test is depicted (*p < 0.05; **p < 0.01; ***p < 0.001; ****p < 0.0001).

To test the *in vivo* protection of the IN trivalent NDV-HXP-S vaccine, Golden Syrian hamsters were vaccinated at a final dose of 10^6^ EID_50_ to observe greater differences between formulations in an *in vivo* challenge model (**Figure 4a**), as previously shown (21). Four different vaccination regimens were compared as shown in **Figure 4b**: live monovalent NDV-HXP-S given IN or IM and live trivalent NDV-HXP-S given IN or IM. A group vaccinated IN with WT NDV was included as negative control. Hamsters were vaccinated in a 12-week interval to allow for the immune response to plateau as shown in **Figure 3**. Twelve weeks after the second boost, hamster serum samples were collected. Ancestral spike-specific (**Figure 4c**), Omicron BA.1 spike-specific (**Figure 4d**) and RBD-specific (**Figure 4e**) antibody titers, and neutralizing antibody titers against ancestral (**Figure 4f**) and Omicron BA.1 (**Figure 4g**) were measured. While both IN live monovalent and trivalent NDV-HXP-S vaccines elicited the highest levels of binding-antibody titers (**Figure 4c-e**), trivalent formulations provided more cross-reactive neutralizing antibody titers against Omicron BA.1 than the monovalent ones (**Figure 4g**).

Hamsters were next challenged with Omicron BA.1 strain and throat swab samples were taken 1, 3 and 5-days post challenge to monitor viral oral shedding (**Figure 4h**). Among the different regimens tested, IN trivalent NDV-HXP-S vaccine showed the fastest elimination of Omicron BA.1 viral copies in the throat (**Figure 4h**, dark pink dotted line, p value<0.05 versus. NDV WT group at 3 and 5 dpi), followed by IN monovalent vaccine (**Figure 4g**, dark blue dotted line), again demonstrating the advantages of the mucosal vaccine at preventing viral shedding. The live trivalent vaccine given IM (**Figure 4h**, dark pink solid line) also reduced oral viral copies more efficiently than the monovalent vaccine given IM (**Figure 4h**, dark blue solid line). The lowest level of throat viral copies in animals IN vaccinated with the live trivalent vaccine correlated with the absence of infectious virus in nasal turbinate measured by plaque assay (**Figure 4i**). Infection was detected in the other vaccinated groups being the highest in animals given IM monovalent NDV-HXP-S vaccine. No infectious viruses were detected in the lungs of vaccinated animals (**Figure 4j**). Collectively, these data show that the presence of mucosal immunity provided by IN vaccination and the elicitation of cross-reactive antibodies by the trivalent formulation additively contribute to reduce Omicron BA.1 in the upper respiratory tract. Overall, an improved *in vivo* protection against Omicron BA.1 infection was observed with a live trivalent NDV-HXP-S vaccine formulation, administered intranasally or intramuscularly, in that intranasal vaccination is more effective than intramuscular vaccination.

### Immune responses of the trivalent vaccine as a third booster in mice

**Figure 5:**
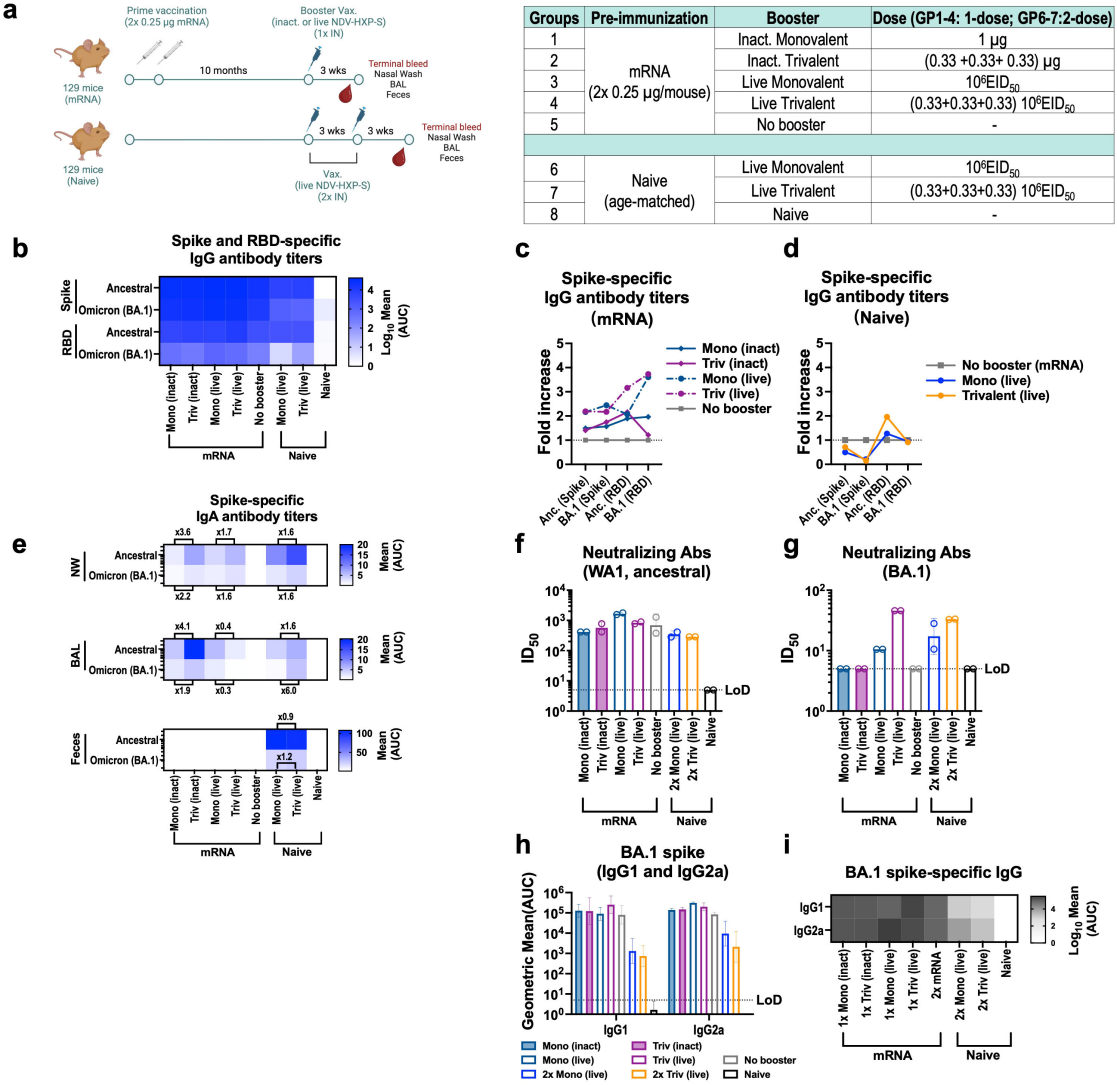
Intranasal live NDV-HXP-S vaccines boost humoral and mucosal immunity after two doses of 0.25 µg mRNA in mice. **(a)** Experimental design and vaccination groups. Eight- to ten-week-old female 129 mice were vaccinated with two doses 0.25 µg of Comirnaty mRNA vaccine (Pfizer) and 10 months later, mice were intranasally (IN) boosted with either 1 µg of inactivated or 10^6^ EID_50_ of live monovalent or trivalent NDV-HXP-S vaccine. Naïve age-matched mice were vaccinated in a two-dose regimen with 10^6^ EID_50_ total dose of monovalent or trivalent NDV-HXP-S vaccine as positive vaccination controls and a no booster group and complete naïve mice were used as negative controls. Blood serum, nasal washes, BAL, feces and spleen were harvested 3-weeks after the boost. **(b)** Heatmap of mean spike and RBD-specific IgG serum antibody titers and **(c-d)** fold-increase in serum antibody titers of boosted groups over no booster control against ancestral (Wuhan) or Omicron BA.1 proteins (n=4) measured by ELISAs 21 days after last boost. Inactivated groups are shown in continuous lines whereas live are shown in discontinuous lines in Fig **(c)** The GMT of fold increase of serum antibody titers over two doses 0.25 µg (grey squares) of Comirnaty mRNA vaccine (Pfizer) are depicted. **(e)** Heatmap of mean spike-specific IgA antibody titers against ancestral (Wuhan) or Omicron BA.1 spike (n=4) measured 21 days after last boost by ELISAs in nasal washes (NW), bronchioalveolar lavage (BAL) and feces samples. **(f-g)** Neutralizing activity of post-boost pooled sera was tested in microneutralization (MNT) assays against USA-WA1/2020 strain, and Omicron BA.1variant in technical duplicates. GMT serum dilutions inhibiting 50% of the infection (ID_50_) is plotted (limit of detection equals to 10 and a value of 5 and was assigned to negative samples). GMT ID_50_ ± SD are depicted. **(h-i)** Omicron BA.1 serum IgG1 and IgG2a antibody titers (n=3-4).

By the third quarter of 2022, ninety six percent of the population older than 16 years had SARS-CoV-2 antibodies from previous infection or vaccination (35). Despite of this high pre-existing immunity, the emergence of new VOCs, like XBB.1.5, JN.1 or KP.2 which evade previous immunity, still poses a public health problem in the post-pandemic era. We evaluated whether a trivalent NDV-HXP-S vaccine would be able to boost the same cross-reactive immune response in the context of mass pre-existing immunity. To do so, 8- to 10-week-old 129 mice were vaccinated with two doses of Comirnaty (Pfizer BioNTech) mRNA vaccine at a dose of 0.25 µg per mouse to reflect real world scenario in human populations. The 129-mouse model has shown great promise to characterize SARS-CoV-2 immunogenicity (20, 36). Ten months later, we vaccinated them with either inactivated NDV-HXP-S given IM (**Supplementary Figure S4A**) or live/inactivated NDV-HXP-S given IN (**Figure 5a**). Naïve age-matched mice were vaccinated in a 2-dose regimen of monovalent or trivalent NDV-HXP-S vaccine as positive vaccination controls. A no booster control group and naïve mice negative control group were included. For pre-immune mice, three-weeks after the booster, animals were euthanized, and spike-specific antibody levels were measured. A boost in ancestral and Omicron BA.1 spike-specific and RBD-specific antibody titers, was obtained with the IM inactivated vaccine boosters (**Supplementary Figure S4C**). This increase correlated with enhanced neutralizing antibody titers against both viruses, proving the capacity of NDV-HXP-S vaccine to work as a third booster (**Supplementary Figure S4E**). However, in any case, the trivalent booster showed advantage (even less) over the ancestral vaccine at inducing cross-reactive antibodies against BA.1. This suggested that a strong immune imprinting by the ancestral mRNA vaccine might restrain the *de novo* Omicron-specific responses induced by the trivalent vaccines in the model tested.

Next, IN booster vaccines were compared in the same model (**Figure 5**). Four formulations were tested: inactivated monovalent, inactivated trivalent NDV-HXP-S vaccine and their live-vectored version, all administered IN. We included the inactivated versions to examine the contribution of the live nature of the vector to the vaccine humoral, mucosal and cellular immunity (**Figure 5a**). Three weeks after the booster, spike-specific and RBD-specific serum antibodies against ancestral Wuhan and Omicron BA.1 were measured, shown as a heatmap (**Figure 5b**) and fold-increase of titers over no booster group was calculated (**Supplementary Figure S5C-D**). IN live boosters presented a higher increase in serum IgG antibody titers against the ancestral and BA.1 spike/RBD (**Figure 5c**, dark blue and dark pink dotted lines) than IN inactivated vaccines (**Figure 5c**, dark blue and dark pink solid lines). The induction of mucosal immunity after the booster was next measured in the upper and lower respiratory tract and in the gut by collecting nasal washes (NW), BAL fluid (BALF) and feces, respectively (**Figure 5e**). mRNA immunized mice without the booster showed no spike-specific IgA antibodies whereas mice receiving any of the four IN boosters developed spike specific IgA titers in both nasal washes and BALF. Interestingly, inactivated vaccines showed higher IgA values than the live versions. This result needs to be explored in future studies to understand its biological relevance. Of note, in contrast to the IM inactivated booster in **Supplementary Figure S4F**, live trivalent NDV-HXP-S vaccine induced more Omicron BA.1 neutralizing antibody titers than the live monovalent vaccine (**Figure 5g**), while both formulations induced marginal increase of neutralizing antibodies against the ancestral virus (**Figure 5f**). This suggested a mucosal booster after a systemic primary vaccination might be able to generate *de novo* antibody responses. Finally, IgG antibody subclasses were examined (**Figures 5h-i**), where a boost in these two subclasses was observed for all boosters, with the highest one reported in the live trivalent group.

Cellular immunity was measured by MHC-I spike-specific VNFNFNGL-tetramer staining of peripheral blood and intracellular cytokine staining (ICS) of splenocytes 3 weeks after the booster (**Supplementary Figure S5**). For ICS, ancestral and Omicron BA.1 spike-peptide pool stimulated polyfunctional T cells expressing interferon-γ (IFN-γ), tumor necrosis factor-α (TNF-α) and interleukne-2 (IL-2) were compared. Mice vaccinated with two doses of mRNA developed antigen-specific CD4^+^ and CD8^+^ T cells (**Supplementary Figure S5A-D**), as well as circulating SARS-CoV-2 tetramer-specific CD8^+^ T cells (**Supplementary Figure S5E**). A recall of polyfunctional CD4^+^ T cells was observed for all the booster formulations to various degrees among which the live monovalent vaccine induced the most robust response (**Supplementary Figure S5A, C**). The live monovalent vaccine also increased polyfunctional CD8^+^ T cells more predominantly than the other groups (**Supplementary Figure S5B, D**).

## Discussion

NDV has proven to be a safe viral vector vaccine in several preclinical and clinical studies (5–8, 11, 37). Here, biodistribution of the avirulent live NDV LaSota vector has shown to be limited to the site of administration in mice and hamsters. As an avian paramyxovirus, NDV predominantly enters cells by binding to cell receptors with terminal 2,3α sialic acids (38, 39). Presence of 2,3α sialic acid has been described in several cell types in the respiratory airways of BALB/c mice, Golden Syrian hamsters, and humans, which support the NDV entry in those tissues (38, 40–42). Despite this restricted replication, the live NDV-HXP-S proved to be highly immunogenic even after only one dose in mice and hamsters. Furthermore, it was able to reduce SARS-CoV-2 viral shedding in the upper respiratory tract when given IN. This proof of concept shows great promise toward the use of NDV-HXP-S to reduce SARS-CoV-2 transmission in high-risk populations like the elderly and people with comorbidities for whom a faster decay of antibodies after prime vaccination has been reported (43–45).

Aiming to improve the breadth of protection, we optimized a trivalent formulation combining the ancestral, Beta and Delta spikes, which previously had shown to induce better neutralizing antibodies than the ancestral monovalent vaccine against mismatched VOIs, using IM vaccination (14). Here, we investigated the immune responses induced by the live multivalent vaccine with the same variant components. We demonstrated that the live IN trivalent NDV-HXP-S vaccine was superior at generating cross-neutralizing antibodies against Omicron BA.1 over the ancestral monovalent vaccine in the two animal models tested. These cross-reactive antibodies seem to target the RBD region (i.e. higher BA.1 RBD-binding antibodies with trivalent vaccines) (46).

Due to rapid evolution of the SARS-CoV-2 variants and their immune evasion, several health agencies have recommended booster vaccinations to be changed to match the circulating variant (47–49). In this work, we explored the use of a broadly cross-reactive multivalent vaccine as an alternative to strain-specific boosters. In mRNA vaccinated mice via the intramuscular route, we saw no difference between the ancestral NDV-HXP-S vaccine versus the trivalent formulation when the inactivated vaccine booster was given IM, likely due to immune imprinting (50, 51). On the other hand, the IN administration of the live vaccine appeared to show a different stimulation of the humoral immune responses that might induce *de novo* variant-specific antibodies (25). Previously, we observed that an Omicron NDV-HXP-S IN booster could induce a higher level of antibodies against the Omicron variant, than the ancestral NDV-HXP-S, in ancestral mRNA vaccine pre-immune mice (25). These two studies combined suggest that a mucosal booster might better overcome the immune imprinting barrier induced by a previous systemic vaccine than a systemic booster. But future studies are imperative to validate such a hypothesis.

Overall, these results provided proof of concept for the development of mucosal multivalent NDV-HXP-S as next-generation COVID-19 boosters. Nonetheless, this study has several limitations. Preclinical studies in mice and hamsters may present a different immunogenic response than those expected in humans. The mechanisms of the difference between 1-dose and 2-dose intranasal live trivalent vaccination remain to be investigated. In addition, future studies investigating multivalent NDV-HXP-S formulations including more recent variants will be necessary to further extend the cross-protection against new variants like JN.1 or KP.2.

## Data Availability

The raw data supporting the conclusions of this article will be made available by the authors, without undue reservation.
