# Supplementary material for: Mucosal multivalent NDV-based vaccine provides cross-reactive immune responses against SARS-CoV-2 variants in animal models
Source: Front Immunol. 2025 Mar 17;16:1524477. doi: 10.3389/fimmu.2025.1524477 (PMC11955676; doi:10.3389/fimmu.2025.1524477)
Supplement: Supplementary file 1 [file DataSheet1.pdf]

Supplementary Material

Mucosal multivalent NDV-based vaccine provides superior cross-reactive immune responses against SARS-CoV-2 variants in animal models

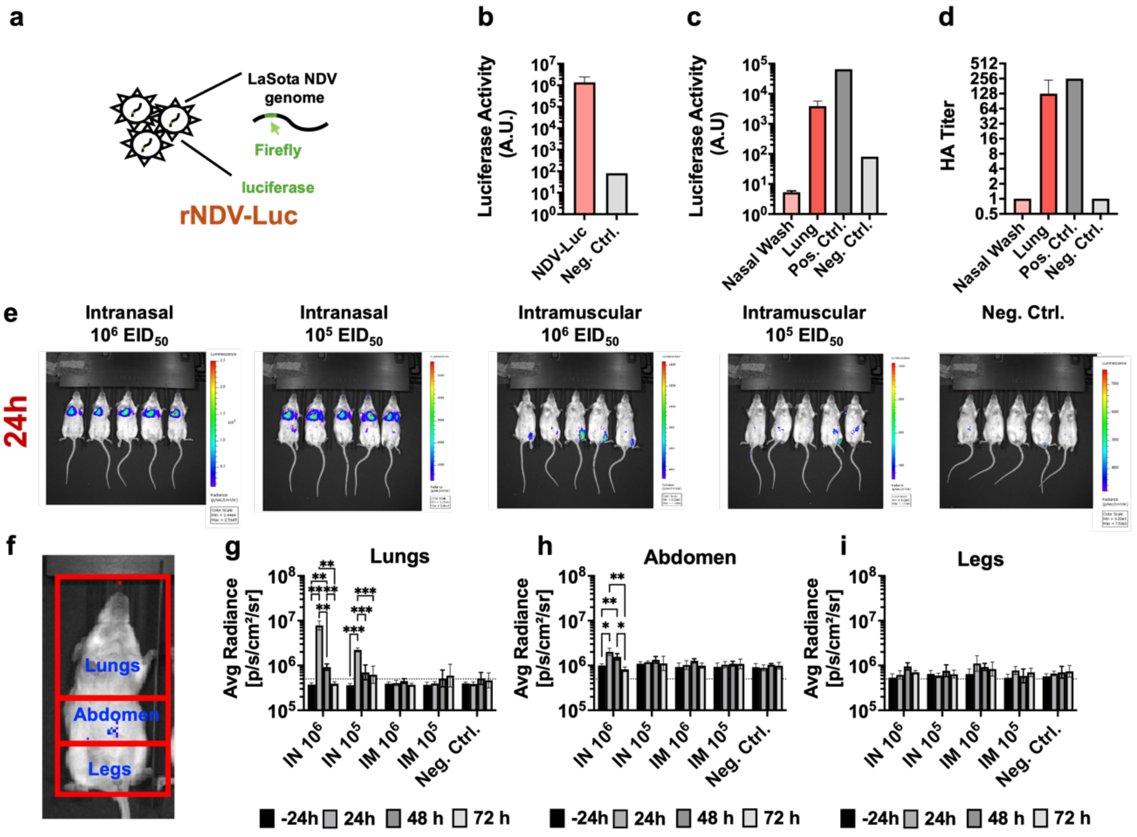

Supplementary Figure 1. Development of a reporter NDV vector for *in vivo* biodistribution studies. (a) Development of a mouse codon optimized reporter NDV vector expressing FireFly luciferase (rNDV-luc). FireFly luciferase gene was introduced between the *P* and the *M* genes of La Sota NDV and the recombinant virus was rescued by reverse genetics. rNDV-luc activity was confirmed **(b)** *in vitro* and **(c-d)** *in vivo.* **(b)** Luciferase activity of cell lysates from BSRT-7 cells infected (n=3) with rNDV-luc at 48 hours post-infection. BALB/C mice (n=3) were administered 10^6 EID_{50} intranasally in a total volume of 30 µL and 24 hours after infection the nasal wash and lung homogenates were collected and **(c)** luciferase activity and **(d)** HA titer were measured. Recombinant luciferase (n=1) and HA-positive NDV virus stock (n=3) were used as positive controls, respectively. Negative controls are uninfected cell BSRT-7 cell lysates in (c) and PBS in (d), respectively. **(e-i)** *In vivo* biodistribution of rNDV-luc. rNDV-luc was administered to BALB/c mice (n=5) intranasally or intramuscularly at a dose of 10^6 EID_{50} or 10^5 EID_{50} and luciferase activity was measured at 24, 48 and 72 hours post administration using a Biophotonic IVIS© Spectrum *in vivo* imaging system. **(e)** 2D optical tomography of BALB/C mice 24 hours after administration. **(f)** Three different regions of interest were defined to quantify the luminescence: **(g)** lungs, **(h)** abdomen and **(i)** legs. One day prior to administration (-24h), the background bioluminescence was measured as the reference value. The

p values and geometric mean (GMT) of average radiance and two-way ANOVA corrected for Tukey's multiple comparisons test are depicted (*p < 0.05; **p < 0.01; ***p < 0.001; ****p < 0.0001).

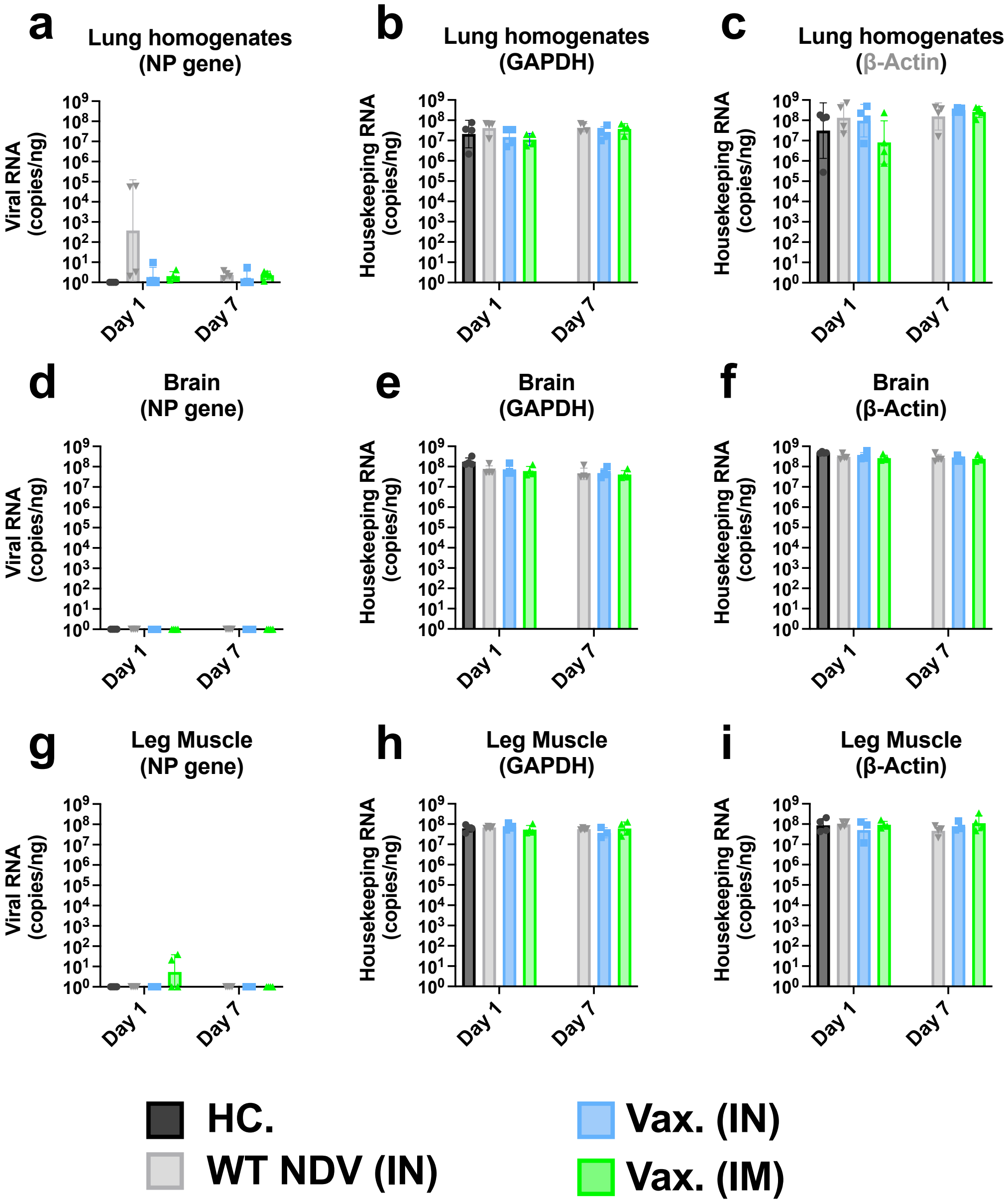

Supplementary Figure 2. Biodistribution of viral RNA from live NDV-HXP-S vaccination in Golden Syrian hamsters measured by RT-qPCR. Golden Syrian hamsters (n=4) were immunized with a total dose of 10^7 EID_{50} of ancestral Wuhan NDV-HXP-S via the IN or IM route. Two more groups vaccinated IN with wild type LaSota (WT) NDV and PBS were added as controls. On day 1 and day 7 after vaccination, lung homogenates (LH), brain and leg muscle at the site of vaccination were collected. Brain and leg muscle were stored in formaldehyde-fixed parafilm embedded (FFPE) blocks from where scrolls were cut, and RNA was extracted. Three sets of primers targeting three different genes were used: NP gene to detect the presence of NDV, and β-actin and GAPDH as housekeeping genes. Viral RNA in copies/ng in **(a)** LH, **(d)** brain and **(g)** leg muscle are depicted.

Housekeeping genes RNA copies/ng in **(b-c)** LH, **(e-f)** brain and **(h-i)** leg muscle for β-actin and GAPDH. An RT-qPCR was run for all samples for each set of primers and the copies/mL were interpolated from the standard curve, then, results were normalized to copies/ng. The melt curve and no template control (NTC) samples were used as quality controls. For samples below the LoD an arbitrary value of 1 was given. GMT ± SD are graphed.

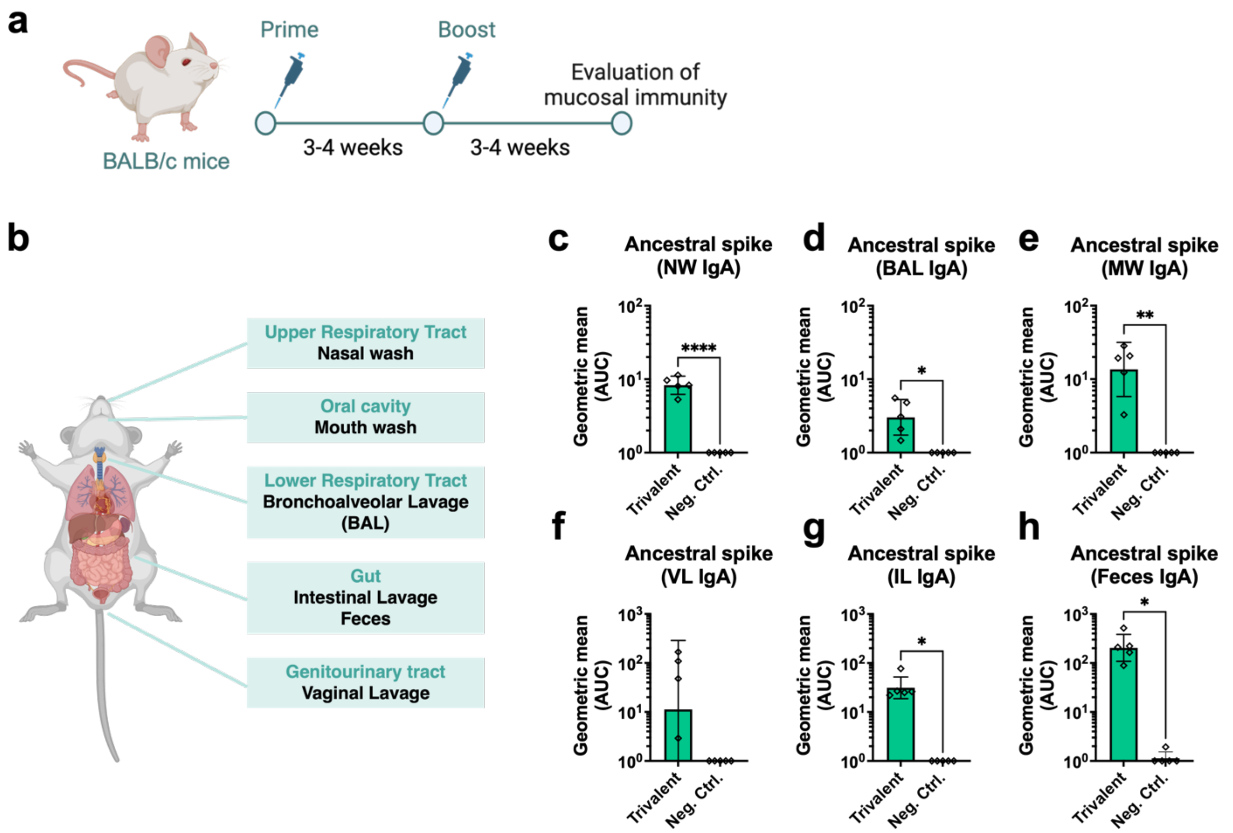

Supplementary Figure 3. Intranasal vaccination of live trivalent NDV-HXP-S induces mucosal immunity. (a) Experimental design. Eight- to ten-week-old female BALB/c mice were vaccinated with 10^6 EID_{50} of live trivalent NDV-HXP-S variant vaccines (Trivalent IN) or WT NDV (negative control). Two immunizations were performed via the intranasal route (IN) with a three-week interval. **(b-h)** Mucosal antibodies induced by live trivalent NDV-HXP-S vaccine. **(b)** Three to four weeks after the second boost, several mucosal fluids were collected and spike-specific IgA titers against ancestral (Wuhan) spike (n=5) in **(c)** nasal washes, **(d)** bronchoalveolar lavage (BAL) fluid, **(e)** mouth wash, **(f)** vaginal lavage, **(g)** intestinal lavage, and **(h)** feces were measured. Unpaired two-tailed T test is depicted ($*p < 0.05$; $**p < 0.01$; $***p < 0.001$; $****p < 0.0001$).

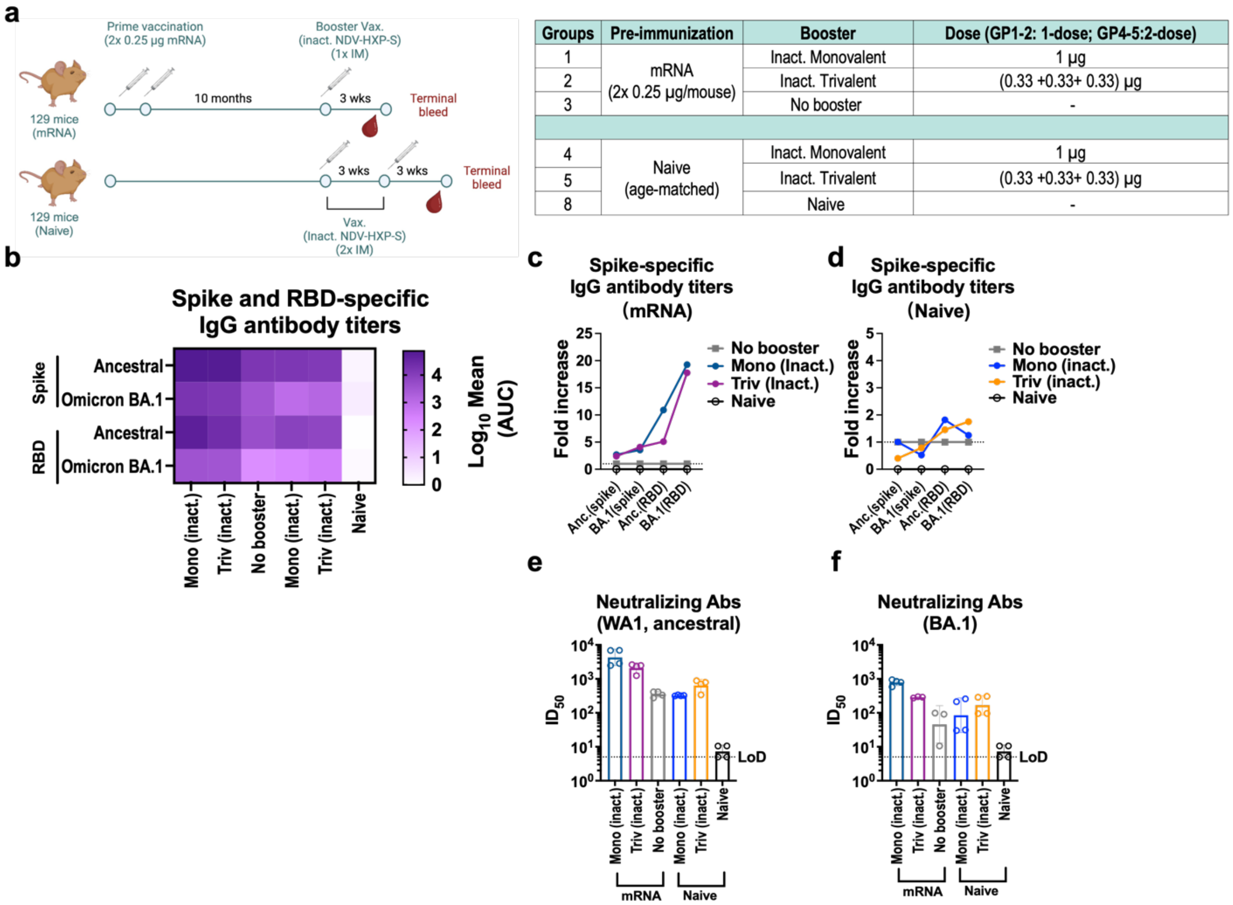

Supplementary Figure 4. Intramuscular vaccination with inactivated NDV-HXP-S vaccines boosts humoral immunity after two doses of 0.25 µg mRNA in mice. (a) Experimental design and groups. Eight- to ten-week-old female 129 mice were vaccinated with 0.25 µg of Comirnaty mRNA vaccine (Pfizer) and 10 months later, mice were boosted IM with either 1 µg of inactivated monovalent or trivalent NDV-HXP-S vaccine. Naïve age-matched mice were vaccinated in a two-dose regimen with 1 µg of monovalent or trivalent NDV-HXP-S vaccine as controls. A no booster group and naïve mice were used as negative controls. **(b)** Heatmap of spike-specific and RBD-specific IgG serum antibody titers and (**c-d**) fold-increase in serum antibody titers of boosted groups over no booster control against ancestral (Wuhan), and Omicron BA.1 (B.1.1.529) proteins (n=4) measured by ELISAs at 21 days after the last boost. The geometric mean of fold increase of serum antibody titers in pre-immune animals receiving an **(c)** NDV-HXP-S booster or **(d)** in naïve animals receiving only 2x doses of NDV-HXP-S over two doses 0.25 µg mRNA vaccinated mice are depicted. **(e-f)** Neutralizing antibody titers . Post-boost pooled sera were tested in microneutralization (MNT) assays against **(e)** USA-WA/2020 strain and **(f)** Omicron (B.1.1.529) variant in technical duplicates. GMT serum dilutions inhibiting 50% of the infection (ID_{50}) were plotted (limit of detection equals to 10 and a value of 5 was assigned to negative samples).

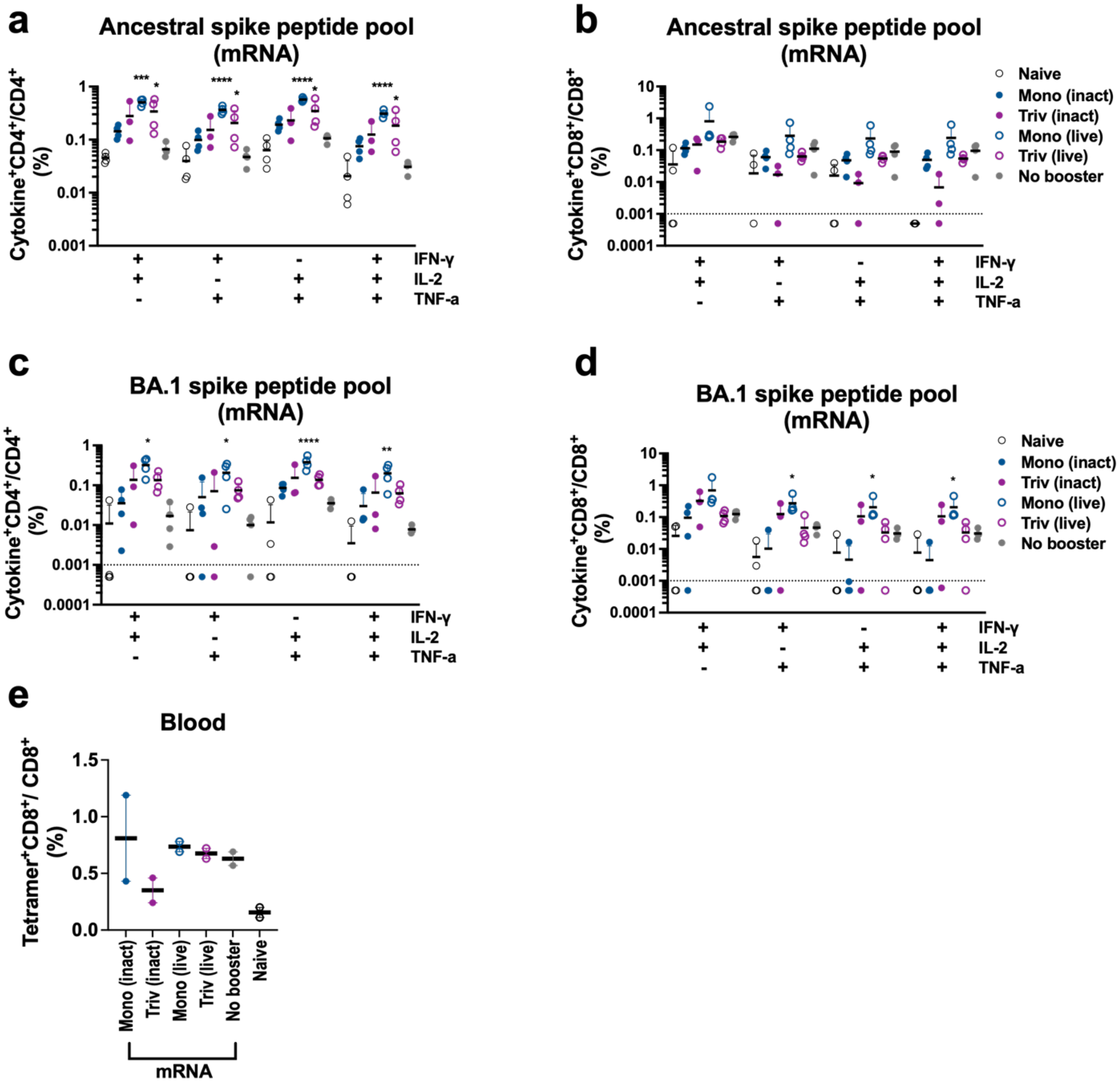

Supplementary Figure 5. Intranasal live NDV-HXP-S vaccination boosts T cell immunity after two doses of 0.25 µg mRNA in mice. Eight- to ten-week-old female 129 mice were vaccinated with two doses of 0.25 µg of Comirnaty mRNA vaccine (Pfizer) and 10 months later mice were boosted either with 1 µg of inactivated or 10^6 EID_{50} of live monovalent or trivalent NDV-HXP-S vaccine given IN. Naïve age-matched mice and no-booster mice were used as controls. Spleens from vaccinated mice were harvested 3-weeks after the boost. **(a-d)** Polyfunctional T cell analysis by intracellular cytokine staining (ICS) in the spleen (stimulated samples relative to unstimulated samples). Splenocytes were *ex vivo* stimulated with Ancestral or Omicron BA.1 spike specific peptide pool and intracellular cytokine staining was performed and analyzed by FACS. Intracellular cytokine staining was used to measure antigen-specific production of IFN-γ, TNF-α and IL-2, by **(a,c)** $CD4^+$ or **(b,d)** $CD8^+$ T cells. **(e)** Spike-specific $CD8^+$T cells in the blood. SARS-CoV-2 spike tetramer-specific $CD8^+$ T cells were

quantified in the blood three-weeks after boosting ($CD3^{+}$ $CD8^{+}$MHC II^{-} $tetramer^{+}$). Blood of individual animals was pooled for each group and analyzed in technical duplicates. Mean ±SD was graphed. One-way ANOVA for each polyfunctional cytokine profile and tetramer+ CD8+ T cells against the no booster group and corrected for Dunnett's multiple comparisons test are depicted (*$p < 0.05$; **$p < 0.01$; ***$p < 0.001$; ****$p < 0.0001$).
